# Targeting Dynamin-Related Protein 1 and Glucose Metabolism Reverses Acquired Resistance to Sorafenib in Liver Cancer

**DOI:** 10.32604/or.2026.067443

**Published:** 2026-06-16

**Authors:** Jinhui Che, Zhiyuan Chen, Feng Zhang, Shuo Zhu, Shengya Cao, Ou Li, Rong Li, Jun Zheng, Yubin Liu

**Affiliations:** 1 Department of Hepatobiliary Surgery, Department of General Surgery, Guangdong Provincial People’s Hospital, Guangdong Academy of Medical Sciences, Southern Medical University, Guangzhou, China; 2 Department of Hepatobiliary Surgery, Xuzhou Cancer Hospital, Xuzhou, China; 3 Department of Hepatic Surgery and Liver Transplantation Center of the Third Affiliated Hospital of Sun Yat-sen University, Guangzhou, China; 4 Guangdong Key Laboratory of Liver Disease Research, Key Laboratory of Liver Disease Biotherapy and Translational Medicine of Guangdong Higher Education Institutes, the Third Affiliated Hospital of Sun Yat-sen University, Guangzhou, China; 5 Department of Clinical Laboratory, Xuzhou Cancer Hospital, Xuzhou, China

**Keywords:** Sorafenib-resistant hepatocellular carcinoma, mitochondrial dynamics, glucose metabolism, mdivi-1, IACS-010759

## Abstract

**Objective:** Advanced liver cancer, a highly lethal and increasingly prevalent malignancy, frequently develops sorafenib resistance, with aberrant mitochondrial dynamics and metabolism implicated in its pathogenesis. This study aimed to investigate their interplay and assess combination therapies against sorafenib-resistant liver cancer. **Methods:** Mitochondrial morphology was assessed using immunofluorescent staining. Besides, the mitochondrial metabolic profile was evaluated by measuring the oxygen consumption rate, glucose uptake, and lactate production. Dynamin-related protein 1 (Drp1) expression was determined through immunohistochemical staining, western blotting, and reverse transcription-quantitative polymerase chain reaction (RT-qPCR). Cell counting, colony formation, and cell cycle assays were conducted to evaluate *in vitro* cell growth. Furthermore, time-lapse cell motility and Transwell assays were employed to assess cell migration and invasion capacities, respectively. Orthotopic xenograft models were utilized to demonstrate the therapeutic effects of the combined administration of the oxidative phosphorylation (OXPHOS) inhibitor IACS-010759 and the Drp1 inhibitor mdivi-1. **Result:** Importantly, our findings revealed that Drp1-mediated mitochondrial fission and the metabolic switch from OXPHOS to aerobic glycolysis were dominant in sorafenib-resistant liver cancer cells and strongly correlated with tumor prognosis (hazard ratio = 3.899, 95% confidence interval: 1.167–13.022, *p* = 0.027). Drp1 knockdown or inhibition impaired the invasive and metastatic capabilities of these cancer cells but promoted cell cycle progression and cellular growth, attributed to a metabolic shift from aerobic glycolysis to OXPHOS. Notably, the combined administration of the OXPHOS inhibitor IACS-010759 with mdivi-1 significantly attenuated tumor progression in sorafenib-resistant liver cancer, affecting both proliferation and metastasis. **Conclusion:** The results of this study collectively indicate that mitochondrial dynamics regulate metabolism in sorafenib-resistant liver cancer, which displays an aggressive hybrid metabolic phenotype. Accordingly, the combined targeting of mitochondrial dynamics and metabolism may represent an effective strategy to overcome sorafenib resistance in liver cancer.

## 1 Introduction

Liver cancer is a highly lethal malignancy and is now ranked as the third most common cause of digestive system cancer-related death worldwide [1]. Although surgical resection, radiofrequency thermal ablation, and liver transplantation are the mainstay of treatment for patients with early-stage liver cancer, approximately 70% of cases are diagnosed at an advanced stage, resulting in poor survival outcomes [2,3]. Therapies targeting cell cycle checkpoints are frequently employed as anti-cancer treatments [4]. Sorafenib, the first-line oral multi-kinase inhibitor, has been reported to improve the survival of patients with advanced-stage liver cancer by approximately three months [5]. Sorafenib directly suppresses cell proliferation by inhibiting the tyrosine kinase (TK) activity of platelet-derived growth factor receptor-beta (PDGFR-β) and vascular endothelial growth factor receptors 1–3 (VEGFR 1–3), as well as the kinase activity of Raf-1 and B-Raf, thereby blocking the RAS/RAF/MEK/ERK signaling pathway [6]. However, only 30% of patients benefit from sorafenib treatment, and acquired drug resistance often develops within six months [7], which is a primary cause of its limited efficacy in liver cancer patients. Multiple factors contribute to sorafenib resistance in this patient population. Wu et al. [8] found that adrenergic receptor beta 2 (ADRB2) signaling could induce sorafenib resistance in a diethylnitrosamine (DEN)-induced hepatocellular carcinoma (HCC) mouse model by suppressing autophagy. Given that sorafenib remains the first-line and cornerstone treatment for advanced HCC, it is crucial to investigate the underlying mechanisms of sorafenib resistance and explore potential novel strategies to enhance its efficacy.

Numerous studies have indicated that mitochondria, the bioenergetic and signaling organelles responsible for cellular growth, apoptosis, autophagy, redox homeostasis, and energy metabolism, play a critical role in carcinogenesis and cancer progression [9]. Mitochondria also exhibit high morphological dynamism, regulated by the opposing processes of fusion and fission, to meet cellular energy demands and adapt to hostile environments characterized by hypoxia and energy stress [10]. The underlying molecular requirements of these two processes are well-established and include dynamin-related protein 1 (Drp1) and fission protein 1 (Fis1) for mitochondrial fission, and mitofusins (MFN1 and MFN2) and optic atrophy 1 (OPA1) for mitochondrial fusion [11]. Most cancer cells, including liver cancer cells, display dysregulated mitochondrial dynamics characterized by hyperactive fission induced by Drp1, resulting in the formation of short, isolated, dot-like spheres [12]. However, the impact of mitochondrial dynamics on liver cancer resistance to sorafenib treatment remains poorly understood.

As the central hub for energy metabolism, mitochondria significantly influence the glucose metabolism of cancer cells, which are often characterized by a pro-glycolytic phenotype or the Warburg effect, exhibiting elevated glucose uptake and lactate production, along with an impaired oxygen consumption rate (OCR) [13]. While considerable efforts have been directed towards therapeutically targeting glycolysis, clinical outcomes have been unfavorable [14]. Previous studies have indicated that specific subpopulations of cancer cells rely on mitochondrial OXPHOS for both bioenergetic and biosynthetic processes, in addition to demonstrating a substantial dependence on glycolysis. This observation underscores the metabolic plasticity of these cell populations [15,16]. Consequently, it was hypothesized that sorafenib-resistant liver cancer would exhibit a more aggressive hybrid metabolic phenotype following the development of drug resistance. Therefore, combination therapies that target this metabolic complexity warrant further investigation.

In this study, we aimed to elucidate the relationship between mitochondrial dynamics and metabolism in sorafenib-resistant liver cancer and to investigate the potential of a combination therapy targeting both pathways.

## 2 Materials and Methods

### 2.1 Patients and Tissue Specimens

This study was conducted in accordance with the Declaration of Helsinki and with the approval of the Ethics Committee of Guangdong Provincial People’s Hospital (Guangzhou, China; approval no KY2020-261-01-01) in 2020. Fifty-six paired liver cancer specimens and corresponding adjacent normal tissues (ANT), along with ten healthy liver tissues (obtained from patients with hepatic benign tumors who underwent curative hepatectomy), were collected at the Department of General Surgery of Guangdong General Hospital (Guangzhou, China). These samples were immediately frozen and stored at −80°C for subsequent screening using reverse transcription-quantitative polymerase chain reaction (RT-qPCR). Besides, 90 patients who received a histological diagnosis and had not undergone any pre-operative therapy, including radiotherapy, chemotherapy, or targeted therapy, were retrospectively enrolled in this study. Paraffin-embedded specimens from these patients were obtained for immunohistochemical (IHC) analysis. Detailed information on the enrolled patients was collected following each surgery. In compliance with the policies of the Ethics Committee, each patient provided written informed consent.

### 2.2 Cell Lines and Culture

The highly aggressive liver cancer cell line HCC-LM3, two lowly aggressive liver cancer cell lines, Huh7 and HepG2, and 293T cells were obtained from the Cell Bank of Type Culture Collection of the Chinese Academy of Sciences (Shanghai, China). All cell lines were authenticated by short tandem repeat (STR) analysis and confirmed to be mycoplasma-free. The cell lines were cultured in high-glucose Dulbecco’s Modified Eagle Medium (DMEM; Gibco; Thermo Fisher Scientific, Inc, Waltham, MA, USA) supplemented with 10% fetal bovine serum (FBS; PAN-Biotech GmbH, Aidenbach, Bavaria, Germany) and maintained in an incubator at 37°C and 5% CO_2_ under mycoplasma-free conditions. To establish sorafenib-resistant clones, Huh7 and HepG2 cells were continuously exposed to gradually increasing concentrations of sorafenib (Selleck Chemicals, Houston, TX, USA) to determine the half-maximal inhibitory concentration (IC_50_) of sorafenib in these cell lines. Cells that remained adherent at the end of the third round of drug treatment were considered resistant. The IC_50_ values were determined to be 10 μM for Huh7 and 7 μM for HepG2. Huh7 and HepG2 cells were treated with their respective IC_50_ concentrations of sorafenib for 72 h, after which the remaining cells were harvested and sub-cultured. This process was repeated for five rounds to generate sorafenib-resistant Huh7 and HepG2 (Huh7-SR, HepG2-SR) cell lines.

### 2.3 Total RNA Extraction and RT-qPCR

Total RNA extraction and RT-qPCR (Table S1 were conducted as previously described [5]). Briefly, cells (Huh7, Huh7-SR, HepG2 and HepG2-SR) and tissues from different groups were harvested, and total RNA was extracted using TRIzol reagent (Invitrogen; Thermo Fisher Scientific, Inc.) according to the manufacturer’s protocol. Subsequently, the extracted RNA was reverse transcribed into cDNA using a Transcriptor First Strand cDNA Synthesis Kit (catalog: 04897030001; Roche Applied Science, Basel, Switzerland) and amplified using a PCR thermal cycler (T100 thermal cycler; Bio-Rad Laboratories, CA, USA) with the following parameters: initial heating at 65°C for 10 min, incubation at 55°C for 30 min, deactivation at 85°C for 5 min, and storage at 4°C. Finally, qPCR was performed using the Roche Applied Science SYBR Green I Master Mix and an LC-480 Real-Time PCR System (Roche Applied Science) following the manufacturer’s instructions. The thermocycling conditions were: initial denaturation at 95°C for 30 s, followed by 40 cycles of denaturation at 95°C for 5 s and annealing/extension at 60°C for 30 s. The expression levels of β-actin were used as an internal control for normalization.

### 2.4 Western Blotting

The western blotting procedure was performed as described previously. Total protein for cells (Huh7, Huh7-SR, HepG2 and HepG2-SR) and tissues from different groups were extracted using RIPA buffer (WB3100, NCM Biotech, Suzhou, China) supplemented with protease inhibitor. The protein samples were then quantified, and equal amounts were loaded. Subsequently, the protein samples were separated by 10% SDS-PAGE (Nanjing KeyGEN Biotech Co., Ltd.) and transferred onto PVDF membranes (MilliporeSigma; Merck KGaA, Darmstadt, Germany). The membranes were then blocked with 5% non-fat milk in Tris-buffered saline with Tween 20 (TBST) for 1 h at room temperature to prevent non-specific antibody binding. Following blocking, the membranes were incubated with the respective primary antibodies overnight at 4°C. After washing three times with TBST, the membranes were incubated with the appropriate secondary antibody for 1 h at room temperature. Finally, the blot signals were visualized using enhanced chemiluminescence with a FluorChem System imager (ProteinSimple, San Jose, CA, USA). The resulting images were analyzed using ImageJ software (National Institutes of Health, V1.8.0.112, Bethesda, MD, USA).

### 2.5 Confocal Microscopy

Following treatment according to the experimental design, the cells (Huh7, Huh7-SR, HepG2, HepG2-SR and HCC-LM3) were fixed with 4% paraformaldehyde (PFA) for 30 min at room temperature. After washing twice with phosphate-buffered saline (1× PBS, pH = 7.2–7.4), the cells were incubated with 5% goat serum for 30 min to block non-specific antibody binding. Subsequently, the cells were incubated with the primary antibody (TOM20; Catalog No. ab186735, Abcam, Cambridge, UK, diluted at 1:200) overnight at 4°C. The cells were then treated with a species-matched secondary antibody conjugated with Alexa Fluor 594 (1:1000 dilution; Life Technologies, Carlsbad, CA, USA) for 1 h in the dark at 4°C. After nuclear counterstaining with 4′,6-diamidino-2-phenylindole (DAPI) for 2 min, the cells were visualized using a Zeiss 800 confocal microscope (Nikon Corporation, Tokyo, Japan). The mitochondrial morphological quantification was performed using ImageJ software (National Institutes of Health, Bethesda, MD, USA; version 1.53).

### 2.6 Flow Cytometry Analysis

Following treatment according to the experimental design, the liver cancer cells (Huh7, Huh7-SR, HepG2 and HepG2-SR) were harvested and stained with an Annexin V/propidium iodide (PI) apoptosis detection kit and 7-aminoactinomycin D kit (catalog: 556547, 559925 BD Biosciences, Franklin Lakes, NJ, USA) for 15 min at room temperature in the dark. The level of apoptosis in the liver cancer cells was determined using an 8-color FACS Calibur flow cytometer (BD Biosciences), and the results were analyzed using FlowJo software (V10.4, Tree Star, Inc., Ashland, OR, USA).

### 2.7 Colony Formation Assay

Liver cancer cells (Huh7, Huh7-SR, HepG2 and HepG2-SR) from different treatment groups were seeded in 6-well plates at a density of 3000 cells per well and cultured in complete medium under standard conditions for 14 days. Subsequently, the cell colonies were fixed with 4% PFA for 30 min at room temperature and stained with Giemsa solution for 10 min. The results were observed using an inverted light microscope (Leica Microsystems GmbH, DM4 B, Wetzlar, Lower Saxony, Germany).

### 2.8 Transwell Invasion Assay

The Transwell invasion assay was performed as previously described [17], and all experiments were independently repeated three times. Briefly, the upper chambers of Transwell plates were pre-coated with Matrigel (Invitrogen; Thermo Fisher Scientific, Inc.). Subsequently, 200 μL of serum-free culture medium containing 2 × 10^3^ cancer cells (Huh7, Huh7-SR, HepG2 and HepG2-SR) was added to the upper chamber, while the lower chamber was filled with complete medium (basal medium supplemented with 10% FBS). Following incubation for 48 h, the cells that had invaded the lower chamber were fixed with 4% PFA for 30 min at room temperature and then stained with 0.2% crystal violet solution. The number of invaded cells was subsequently quantified by counting them under an inverted light microscope (Leica Microsystems GmbH).

### 2.9 Time-Lapse Cell Motility Assay

The migrative capacity of cells in each group was detected by a Zeiss Axio Observer Z1 microscope (AXIO OBSERVER 5, Oberkochen, Baden-Wurttemberg, Germany). Briefly, cells (Huh7, Huh7-SR, HepG2 and HepG2-SR) were planted in an enclosed chamber and cultured at 37°C with a controlled proportion of CO2. Computer-assisted tracking was used to monitor cell migration every 20 min for 6 h and presented as wind-rose plots by integrating the data using Excel software (Microsoft, 2021). Image acquisition was performed using a 10× or 20× objective at pre-determined intervals (e.g., every 20 min) for a duration of 6 h. To ensure data robustness, multiple fields of view (FOV) were selected, and a hardware-based focus lock system was engaged to prevent thermal drift throughout the recording period. Finally, the migration distances of the cells were analyzed.

### 2.10 Measurement of Glucose Uptake and Lactate Accumulation

The levels of glucose uptake and lactate production were measured using commercially available assay kits according to the manufacturers’ instructions (Glucose Uptake Colorimetric Assay Kit and Lactate Assay Kit, Catalog No. MAK083 and No. MAK329, Sigma-Aldrich; Merck KGaA, Darmstadt, Germany). Briefly, Cell lysates (2 × 10^4^ cells/well) were prepared using Extraction Buffer I, followed by freeze-thaw cycling and heat denaturation at 85°C for 40 min to degrade endogenous NAD(P). After cooling, samples were neutralized with Neutralization Buffer II and supernatants were collected. For NADP activity measurement, samples were incubated with Reaction Mix A at 37°C for 1 h. The reaction was terminated by adding Extraction Buffer I and heating at 90°C for 40 min, then neutralized with Neutralization Buffer II. Amplification was performed using Reaction Mix B, and kinetic absorbance was measured at 412 nm using a microplate reader at 37°C.

### 2.11 ATP Production

The concentration of intracellular ATP was detected using an ENLITEN ATP Assay System Bioluminescence Detection Kit (FF2000, Promega Corporation, Madison, WI, USA) following a previously described method. Briefly, cells (Huh7, Huh7-SR, HepG2 and HepG2-SR) were treated according to the experimental design and incubated in high-glucose DMEM containing 10% FBS for 48 h (5 × 10^4^ cells/well). Subsequently, the cells were harvested and extracted using the ATP Assay Buffer (Promega Corporation). After centrifugation for 5 min, the supernatant was mixed with the ENLITEN ATP Assay System Bioluminescence Detection Kit and incubated at room temperature for 30 min in the dark. The optical density of ATP was measured at a wavelength of 570 nm using a Tecan Spark 10M microplate reader (Tecan Group, Ltd., Mannedorf, Switzerland).

### 2.12 Oxygen Consumption Rate (OCR) and Extracellular Acidification Rate (ECAR) Measurement

Cellular metabolic profiles were assessed using the Seahorse XFe 96 Extracellular Flux Analyzer (Seahorse Bioscience, Inc., Billerica, MA, USA) by measuring OCR and ECAR in accordance with the manufacturer’s guidelines.

OCR: Mitochondrial respiratory function was evaluated through a series of sequential pharmacological interventions. Oligomycin (1 μM; MilliporeSigma; Merck KGaA) was added to inhibit ATP synthase and assess proton leak. Maximal respiratory capacity was then determined by titrating the uncoupler FCCP (500 nM, MilliporeSigma; Merck KGaA). Finally, a combination of rotenone (100 nM, MilliporeSigma; Merck KGaA) and antimycin A (1 μM, MilliporeSigma; Merck KGaA) was used to suppress complex I and III activity, thereby measuring non-mitochondrial respiration. All OCR values were normalized to viable cell counts (2 × 10^4^ cells/well).

ECAR: Cells from each experimental condition were seeded at a density of 2 × 10^4^ viable cells per well in 24-well XF cell culture microplates (Seahorse Bioscience). After 16 h of incubation in complete growth medium to allow adherence, the cells were rinsed twice with pre-warmed XF assay medium [DMEM containing 2 mM L-glutamine (pH 7.4 ± 0.1)] and placed in a non-CO_2_ incubator at 37°C for 60 min to stabilize metabolic activity. The XFp Sensor Cartridge was preloaded as follows: Port A contained 80 mM glucose (to stimulate glycolysis), Port B contained 9 μM oligomycin (to inhibit ATP synthase), and Port C contained 1 M 2-deoxy-D-glucose (2-DG, a hexokinase inhibitor). ECAR recordings were performed using the XFe24 Analyzer under controlled atmospheric conditions.

### 2.13 Mitochondrial Respiratory Complex I–V Enzyme Assays

Activities of mitochondrial complex I–V were measured using enzyme assay kits according to the manufacturer’s protocols (Abcam, ab109721, ab109908, ab287844, ab109911 and ab109714). Briefly, fresh mitochondria were isolated using the Mitochondria Isolation Kit (Abcam, ab110170) and resuspended in PBS supplemented with 10% detergent provided in the kits. Protein concentrations of mitochondrial lysates were estimated for the reaction. Enzyme activities were measured by detecting the absorbance at OD 450 nm in triplicate.

### 2.14 Quantification of Mitochondrial DNA (mtDNA)

Primers targeting a subunit of Complex II (succinate-ubiquinone oxidoreductase) were used as probes for the detection of mtDNA. The primer sequences were as follows: forward, 5′-CAAACCTACGCCAAAATCCA-3′, and reverse, 5′-GAAATGAATGAGCCTACAGA-3′. Mitochondrial DNA was extracted using a Mitochondrial DNA Extraction Kit (GMS20023.1, Genmed Scientifics, Inc., Shanghai, China) according to the manufacturer’s instructions. Briefly, Fresh tissue samples were homogenized on ice in sucrose-based buffer (250 mM sucrose, 20 mM HEPES pH 7.4, 1 mM EDTA) using a Potter-Elvehjem homogenizer. Cell debris and nuclei were removed by centrifugation at 800× *g* for 10 min at 4°C, followed by pelleting of mitochondria at 12,000× *g* for 15 min. The mitochondrial pellet was resuspended in DNase I buffer (50 mM Tris-HCl pH 7.5, 10 mM MgCl_2_) and treated with 50 μg/mL DNase I for 30 min at 4°C to digest contaminating nuclear DNA, with reaction termination by addition of 10 mM EDTA. Mitochondria were further purified by sucrose density gradient centrifugation (20%–60% w/v sucrose gradient, 25,000× *g* for 2 h). Purified mitochondria were lysed in SDS-containing buffer (1% SDS, 100 mM NaCl, 10 mM Tris-HCl pH 8.0, 25 mM EDTA pH 8.0) and subjected to phenol-chloroform-isoamyl alcohol (25:24:1) extraction. DNA was precipitated with ice-cold ethanol, washed with 70% ethanol, and resuspended in nuclease-free water. All steps were performed under RNase-free conditions at 4°C unless specified.

### 2.15 Measurement of Mitochondrial Membrane Potential

Mitochondrial membrane potential was evaluated by flow cytometry using the cationic dye JC-1 (MitoProbe, Life Technologies) in accordance with the manufacturer’s instructions. Briefly, the cells were detached by trypsinization, resuspended in 2 mL of pre-warmed PBS containing 2 μL of JC-1, and incubated for 30 min at 37°C in the dark. After three washes with PBS, JC-1 fluorescence intensity was measured using an eight-color FACSCalibur flow cytometer.

### 2.16 H&E

Formalin-fixed, paraffin-embedded tissue sections (4–5 μm) were subjected to H&E staining. Following deparaffinization and rehydration through graded ethanols, sections were stained with Harris hematoxylin for 5–8 min, rinsed, and briefly differentiated in 1% acid ethanol. Bluing was performed in 0.2% ammonia water, after which cytoplasmic counterstaining was achieved with eosin Y for 1–3 min. Finally, sections were dehydrated, cleared in xylene, and mounted with a resinous medium for examination under a bright-field light microscope.

### 2.17 IHC Staining

IHC staining was conducted according to a previous study [18]. Briefly, 4-μm-thick sections were prepared from paraffin-embedded tissue samples, dewaxed, rehydrated, and subjected to antigen retrieval using EDTA buffer at pH 8.0. Following a 1-h incubation with 5% goat serum at room temperature to block non-specific antigen binding, the sections were incubated with the primary antibody [Anti-DRP1 antibody (Catalog No. ab184247, Abcam, Cambridge, UK), diluted at 1:500); Anti-N-Cadherin antibody (Catalog No. ab18203, Abcam), diluted at 1:300; Anti-E-Cadherin antibody (Catalog No. ab231303, Abcam), diluted at 1:300; Anti-Vimentin antibody (Catalog No. ab92547, Abcam), diluted at 1:200] overnight at 4°C. Subsequently, the slides were washed three times with PBS (1× PBS, PH = 7.2–7.4) and treated with the secondary antibody (Dako A; Agilent Technologies, Inc., Santa Clara, CA, USA) at 37°C for 30 min. The sections were then visualized under a light microscope (Leica Microsystems GmbH, DM4 B, Wetzlar, Germany) after development with diaminobenzidine (DAB) (Dako B + Dako C, 50:1). Immunoreactivity scores were determined based on both staining intensity (none, 0; weak, 1; moderate, 2; strong, 3) and the percentage of positively stained cells (0%, 0; 1–24%, 1; 25–49%, 2; 50–74%, 3; 75–100%, 4). The final immunoreactivity score for each sample was calculated by multiplying the intensity score and the extent score, resulting in a range from 0 to 12.

### 2.18 Animals

Male *BALB/c* nude mice (5 weeks old) were purchased from The Biomedical Research Institute of Nanjing University (Nanjing, China) and handled in accordance with the guidelines of The South China Agricultural University for Animal Experimentation. All experimental animals were housed in laminar flow cabinets under specific pathogen-free conditions at the Laboratory Animal Center of South China Agricultural University. The animals had free access to standard laboratory water and food and were maintained in a controlled environment with a 12-h light/dark cycle, 50% humidity, and a temperature of 20°C.

### 2.19 Mouse Xenograft Models

All experimental animal procedures were approved by the Animal Ethics Committee of Guangdong Provincial People’s Hospital (animal ethics approval number: RG2024-047-01). (Guangzhou, China) and were conducted in accordance with the ARRIVE essential 10 guideline. For the subcutaneous xenograft model, 5 × 10^6^ sorafenib-resistant liver cancer cells (Huh7, Huh7-SR, HepG2 and HepG2-SR) were resuspended in Matrigel and subcutaneously implanted into the right flanks of the *BALB/c* nude mice (Ctr and treatment group, n = 5/group). For treatment, sorafenib (Selleck Chemicals) was administered via oral gavage at a dose of 60 mg/kg body weight (BW) in dimethyl sulfoxide (DMSO). Mdivi-1 (10 mg/kg, MedChemExpress, New Jersey, NJ, USA) was administered via intratumoral injection at a dose of 10 mg/kg BW every 3 days, either alone or in combination with IACS-010759 (Selleck Chemicals) at a dose of 10 mg/kg BW, also administered via intratumoral injection every 3 days. Once tumors became palpable, their length and width were measured every 4 days to monitor growth. Tumor volume was calculated using the formula: $Tumor\;volume=\left(tumor\;{width}^{2}\times tumor\;length\right)/2$ , and the maximum allowable tumor volume was 1.5 cm^3^. After 3 weeks of treatment, all mice were euthanized by cervical dislocation. Cessation of breathing, corneal reflex, and heartbeat were confirmed to ensure successful euthanasia, after which the tumors were collected for further investigation. The volume and weight of the excised tumor samples were also recorded.

For the orthotopic xenograft model, 1 × 10^6^ sorafenib-resistant liver cancer cells were resuspended in 20 μL of Matrigel and injected into the livers of nude mice. Following weighing, the mice were anesthetized via intraperitoneal injection of sodium pentobarbital (1% solution, 40 mg/kg). To assess lung metastasis, all nude mice were sacrificed after 7 weeks, as described previously. The lungs, livers, and intrahepatic tumors were harvested and fixed in 4% PFA. Consecutive 4-μm-thick sections were prepared from the paraffin-embedded lung tissues and stained with hematoxylin and eosin (H&E). The number of metastatic lesions in the lungs was determined by two independent pathologists.

### 2.20 Knockdown of Drp1 Expression

Drp1 expression was *knocked down* using short hairpin (sh) RNA expression vectors containing *a* specific sequence targeting the human Drp1 mRNA (5′-CUACUUCCUGAAAACAAC-3′). A negative control shRNA vector was also utilized. The lentiviral vector was mixed with packaging (psPAX2) and envelope (pMD2.G) plasmids (GenePharma Corporation, Shanghai, China) and transfected into 293T cells using Lipofectamine 3000 (Thermo Fisher Scientific, Inc.) according to the manufacturer’s instructions. Viral supernatants were collected after 48 h. Sorafenib-resistant liver cancer cells (Huh7-SR and HepG2-SR, 4 × 10^5^) were seeded into 6-well plates and transduced with either the control or shDrp1 lentiviruses. Transduced cells were selected using puromycin (MilliporeSigma; Merck KGaA).

### 2.21 Analysis of Prognostic Data from the Cancer Genome Atlas (TCGA)

The association between Drp1 expression and patient overall survival (OS) was analyzed using publicly available data from The Cancer Genome Atlas (TCGA) on the GEPIA website (http://gepia.cancer-pku.cn/index.html). Gene expression data and corresponding clinical information for the relevant cancer cohort (s) were downloaded via the Genomic Data Commons Data Portal. Patients were stratified into high- and low-expression groups based on the median expression value of Drp1. Kaplan-Meier survival curves were generated for the two groups, and the statistical significance of differences in OS was assessed using the log-rank test.

### 2.22 Statistical Analysis

All results were presented as individual values or as the mean ± standard error of the mean (SEM) from at least three independent experiments. Statistical comparisons between two groups were performed using Student’s two-tailed *t*-test. The univariate and multivariate Cox proportional hazards models were used to analyze the independent prognostic factors. All statistical analyses were conducted using GraphPad Prism 7 software (GraphPad Software, San Diego, CA, USA). A *p*-value of less than 0.05 (*p* < 0.05) indicated statistical significance.

## 3 Results

### 3.1 Drp1 Is Upregulated in Sorafenib-Resistant Liver Cancer Cells and Is Closely Correlated with Tumor Metastasis and Patient Prognosis

**Figure 1:**
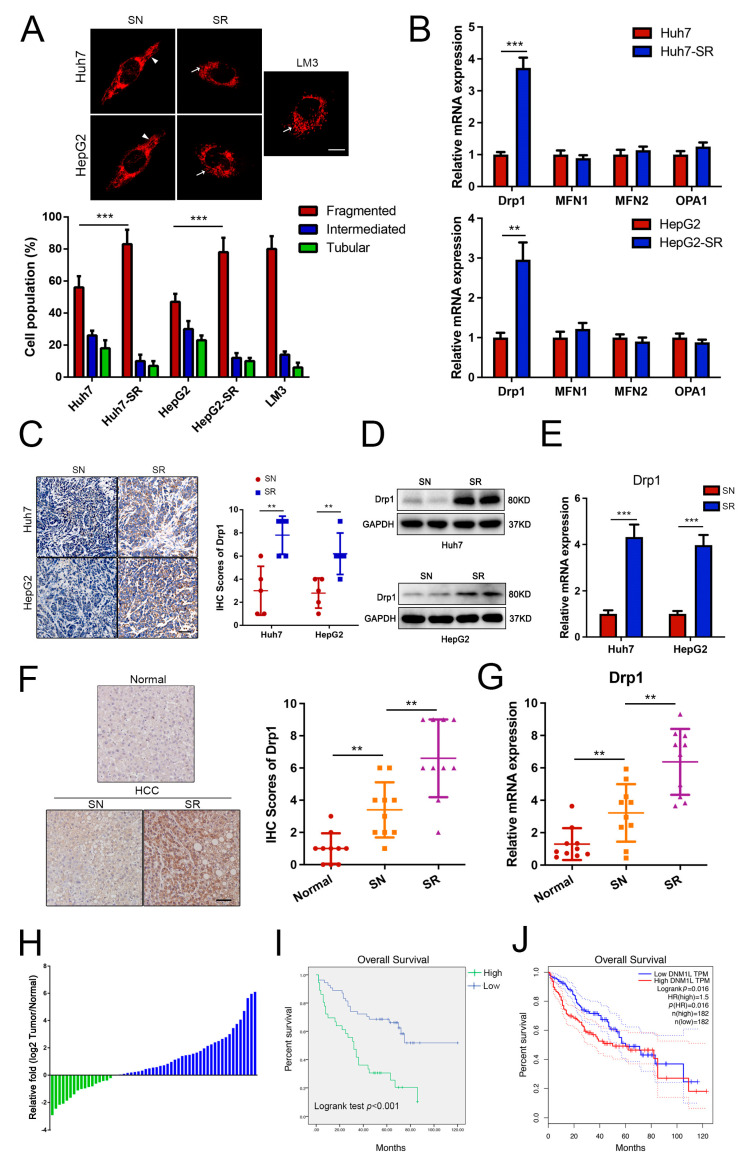
Drp1 is upregulated in sorafenib-resistant liver cancer cells and is closely correlated with tumor metastasis and prognosis. (**A**) Mitochondrial morphology was detected by TOM20 staining (Scale bar: 20 μm). Fragmented mitochondria are marked with arrows and tubular mitochondria are marked with arrowheads. The ratio analysis of the morphological changes of mitochondria of 100 cells per group was calculated based on mitochondrial length. Data are presented as the means ± SEM (n = 3/group). (**B**) The mRNA expression levels of Drp1, MFN1, MFN2 and OPA1 were measured by RT-qPCR. Data are presented as the means ± SEM (n = 3/group). (**C**) IHC staining image of Drp1 (Scale bar: 100 μm) in tumors of sorafenib-resistant and sorafenib-naïve liver cancer cell lines from nude mice. The IHC staining of Drp1 was semi-quantified. Data are presented as the means ± SEM (n = 5/group). (**D**) Western blotting analysis of Drp1. (**E**) The mRNA expression of Drp1 in tumors of sorafenib-resistant and sorafenib-naïve liver cancer cell lines was determined by RT-qPCR. Data are presented as the means ± SEM (n = 5/group). (**F**) Representative images of IHC staining of Drp1 in normal liver and liver cancer tissues from sorafenib-resistant or sorafenib-sensitive patients. Scale bar: 100 μm. The IHC staining of Drp1 was semi-quantified. Data are presented as the means ± SEM. (n = 10/group). (**G**) The mRNA expression of Drp1 in normal liver and liver cancer tissues from sorafenib-resistant or sorafenib-sensitive patients was detected by RT-qPCR. Data are presented as the means ± SEM (n = 10/group). (**H**) RT-qPCR analysis of Drp1 expression in 56 pairs of liver cancer, which showed that Drp1 was significantly upregulated in 70% of HCC patients. The *y*-axis represents relative expression (log^2^ Tumor/Normal). (**I**) Kaplan–Meier analysis of correlations between the Drp1 expression levels and OS of 90 liver cancer patients. (**J**) Kaplan–Meier analysis of correlations between the Drp1 expression levels and OS of 364 liver cancer patients from the TCGA database using the GEPIA website (http://gepia.cancer-pku.cn/index.html). **p < 0.01, ***p < 0.001 (all *p* values were obtained by two-sided Student’s *t*-test). *SR*, sorafenib-resistant; *SN*, sorafenib-sensitive; *HCC-LM3*, Hepatocellular Carcinoma-Lung Metastasis 3.

Previous studies have demonstrated the involvement of mitochondrial dynamics in drug resistance during tumor treatment [19]. To investigate this in the context of sorafenib resistance, we compared the mitochondrial morphology in parental liver cancer cells with that of sorafenib-resistant liver cancer cells (Huh7-SR and HepG2-SR) using the mitochondrial outer membrane protein 20 (TOM20) as a marker. Immunofluorescence staining revealed that Huh7-SR and HepG2-SR cells exhibited a more fragmented mitochondrial network under sorafenib treatment. Given that HCC-LM3 is reportedly a cell line with intrinsic sorafenib resistance, it was included as a positive control (Fig. 1A). To identify key regulatory factors within the mitochondrial dynamic machinery, we measured the mRNA expression levels of the fission regulator *Drp1* and the fusion regulators *MFN1*, *MFN2*, and *OPA1* using RT-qPCR. *Drp1* showed significant upregulation, suggesting its potential role in the observed mitochondrial fragmentation (Fig. 1B). Furthermore, these sorafenib-resistant and sorafenib-naïve liver cancer cell lines were used to establish subcutaneous xenograft models to validate these *in vitro* findings. Immunohistochemical staining, western blotting and RT-qPCR analysis of the tumor tissues from these models confirmed that *Drp1* protein and mRNA expression levels were significantly upregulated in the sorafenib-resistant tumors (Fig. 1C–E).

Next, we examined healthy liver tissues from hepatic benign tumors and liver cancer samples from sorafenib-sensitive and sorafenib-resistant patients obtained during clinical operations. Consistent with our *in vitro* and *in vivo* findings, immunohistochemical staining and RT-qPCR assays revealed that *Drp1* expression was significantly higher in the sorafenib-resistant tumor tissues compared to both the sorafenib-sensitive tumors and the normal liver tissues (Fig. 1F,G). Subsequently, RT-qPCR analysis was performed to compare *Drp1* expression in 56 paired liver cancers. This analysis demonstrated that 70% (39/56) of HCC tissues exhibited high Drp1 expression (Fig. 1H). In a retrospective analysis of 90 patients with liver cancer, correlation analysis of *Drp1* expression in liver cancer tissues (assessed by IHC) and the corresponding patients’ clinical information demonstrated a positive correlation between *Drp1* expression and both tumor diameter and vascular invasion (Table 1). Furthermore, Kaplan-Meier survival analysis revealed that patients with high *Drp1* expression had a significantly poorer prognosis in terms of overall survival, a finding that was further validated using the Cancer Genome Atlas (TCGA) database (Fig. 1I,J). Moreover, multivariate Cox regression analysis demonstrated that *Drp1* expression was an independent predictor for liver cancer (hazard ratio, 3.899; 95% confidence interval, 1.167–13.022; *p* = 0.027; Table 2). These collective results suggest that sorafenib-resistant liver cancer cells exhibit upregulated *Drp1* expression and fragmented mitochondria, which are associated with poorer prognostic outcomes in patients with liver cancer following curative hepatectomy.

**Table 1:** Correlation between Drp1 expression and clinicopathological characteristics in 90 patients with liver cancer.

*Clinicopathological* *Characteristics*		Low Drp1 Group		High Drp1 Group		*p Value*
		N	%	N	%	
Sex						0.605
	Male	51	94.4	33	91.7	
	Female	3	5.6	3	8.3	
Age						0.659
	≤50	20	37.0	15	41.7	
	>50	34	63.0	21	58.7	
AFP						0.469
	≥400	17	31.5	14	38.9	
	<400	37	68.5	22	61.1	
TMN stage						0.001
	III	28	51.9	31	86.1	
	I&II	26	48.1	5	13.9	
Tumor capsule						0.659
	Yes	20	37.0	15	41.7	
	No	34	63.0	21	58.3	
Differentiation						0.227
	Well/Moderation	28	51.9	14	38.9	
	Poor	26	48.1	22	61.1	
Vascular invasion						0.003
	Yes	35	64.8	12	33.3	
	No	19	35.2	24	66.7	

**Note:** *Drp1*, dynamin-related protein 1; *AFP*, alpha-fetoprotein.

**Table 2:** Univariate and multivariate Cox regression analysis of prognostic factors for overall survival in 90 patients with liver cancer.

*Variable*	*Univariate Analysis*			*Multivariate Analysis*		
	*p*	HR	95% CI	*p*	HR	95% CI
Age (years) (>50 versus ≤50)	0.788	0.851	0.262–0.2.762	/	/	/
Sex (Female versus Male)	0.096	0.109	0.008–1.485	/	/	/
HBsAg (Yes versus No)	0.551	0.645	0.152–2.730	/	/	/
AFP (<400 versus ≥400)	**0.013**	**0.162**	**0.038–0.683**	0.022	0.205	0.053–0.797
TNM stage (III versus I&II)	0.940	0.951	0.258–3.508	/	/	/
Tumor capsule (Yes versus No)	0.280	1.999	0.570–7.012	/	/	/
Differentiation (Poor versus Well/Moderate)	**0.021**	**4.621**	**1.266–16.867**	0.017	4.450	1.313–15.089
Vascular invasion (Yes versus No)	**<0.001**	**18.718**	**4.285–81.770**	<0.001	17.646	4.570–68.141
Drp1 expression (Low versus High)	**0.025**	**4.415**	**1.209–16.123**	0.027	3.899	1.167–13.022

**Note:** *Drp1*, dynamin-related protein 1; *AFP*, alpha-fetoprotein; *HR*, hazard ratio. Bold font indicates statistical significance.

### 3.2 Sorafenib-Resistant Liver Cancer Cells Exhibit a Hyperglycolytic Phenotype

**Figure 2:**
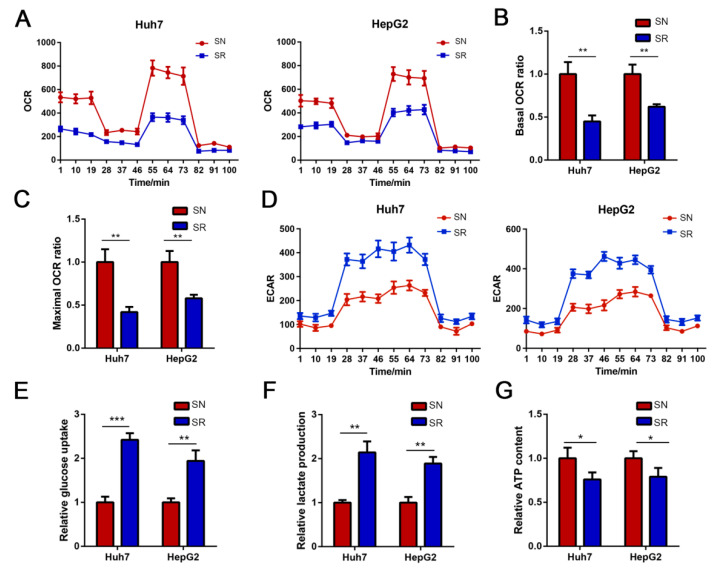
Sorafenib-resistant liver cancer cells are characterized by a hyperglycolytic phenotype. (**A**) The oxygen consumption rate in both sorafenib-resistant and sorafenib-naïve liver cancer cells was measured. (**B**,**C**) Statistical analysis for basal OCR ratio and maximal OCR ratio. Data are presented as the means ± SEM (n = 3/group). (**D**) ECAR in both sorafenib-resistant and sorafenib-naïve liver cancer cells was measured. (**E–G**) The level of glucose uptake, lactate production and ATP content was detected. Data are presented as the means ± SEM (n = 3/group). *p < 0.05, **p < 0.01, ***p < 0.001 (all *p* values were obtained by two-sided Student’s *t* test). *SR*, sorafenib-resistant; *SN*, sorafenib-sensitive.

Mitochondria serve as the central hub for energy metabolism, and their dynamics have a significant impact on glucose metabolism. A characteristic feature of many tumor cells is the preferential utilization of aerobic glycolysis to meet their biosynthetic and bioenergetic demands. Therefore, this study investigated the effects of sorafenib on glucose metabolism in liver cancer cells. Mitochondrial respiration was assessed by measuring the cellular OCR in two sorafenib-resistant and two sorafenib-naïve liver cancer cell lines (Fig. 2A). The results demonstrated a decrease in both basal OCR (Fig. 2B) and maximal respiration levels (Fig. 2C) following the development of sorafenib resistance. Furthermore, ECAR (Fig. 2D), glucose consumption (Fig. 2E) and lactate production (Fig. 2F) were elevated, while ATP content (Fig. 2G) was reduced in sorafenib-resistant liver cancer cells compared to their sorafenib-naïve counterparts. These findings indicate that sorafenib could influence mitochondrial biological activities, leading to a hyperglycolytic phenotype in resistant cells.

### 3.3 Drp1 Promotes Sorafenib-Resistant Liver Cancer EMT and Metastasis via Mitochondrial Dynamic Machinery in Vitro and in Vivo

**Figure 3:**
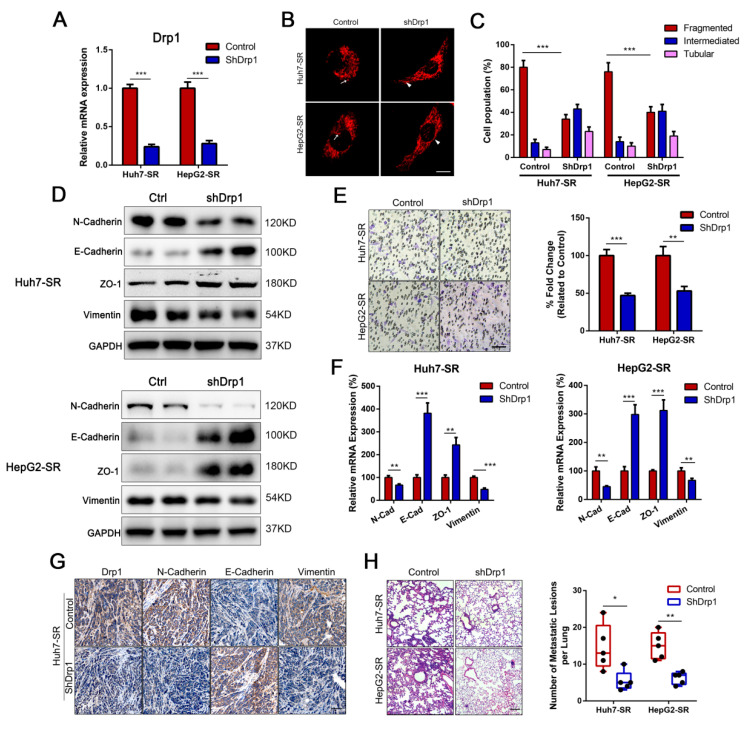
Drp1 promotes sorafenib-resistant liver cancer epithelial-mesenchymal transition (EMT) and metastasis via mitochondrial dynamic machinery *in vitro* and *in vivo*. (**A**) The mRNA expression of Drp1 in two groups was determined by RT-qPCR. Data are presented as the means ± SEM (n = 3/group). (**B**,**C**) Mitochondrial morphology was detected by TOM20 staining (Scale bar: 20 μm). Fragmented mitochondria are marked with arrows and tubular mitochondria are marked with arrowheads. The ratio analysis of the morphological changes of mitochondria of 100 cells per group was calculated based on mitochondrial length. Data are presented as the means ± SEM (n = 3/group). (**D**) Western blotting showed the levels of EMT-related protein in each group of Huh7-SR and HepG2-SR tumors. (**E**) Representative images of Huh7-SR and HepG2-SR cells invasion in each group in a Transwell system (Left) (Scale bar: 100 μm). Statistical analyses of the number of invasive cells (Right). Data are presented as the means ± SEM (n = 3/group). (**F**) The mRNA levels of EMT-related genes (including N-cadherin, E-cadherin, ZO-1 and Vimentin) in two groups were detected by RT-qPCR. Data are presented as the means ± SEM (n = 3/group). (**G**) An orthotopic xenograft model was constructed by implanting Huh7-SR and HepG2-SR cells, respectively. Both primary tumor and the metastatic tumors in the lung were collected. Representative images of immunohistochemical staining of EMT-related genes in primary liver cancer tumors were obtained. Scale bar: 100 μm. (**H**) Representative images of H&E staining of lung tissues. Scale bar: 100 μm (Left). The number of metastatic lesions per nude mouse was calculated (Right). Data are presented as the means ± SEM (n = 5/group). *p < 0.05, **p < 0.01, ***p < 0.001 (all *p* values were obtained by two-sided Student’s *t*-test).

Next, both *in vitro* and *in vivo* loss-of-function experiments were conducted to elucidate the roles of *Drp1* and the mitochondrial dynamic machinery in sorafenib-resistant liver cancer cells. First, Huh7-SR and HepG2-SR stable cell lines with *Drp1* knockdown were established, and RT-qPCR was used to confirm the efficiency of gene silencing (Fig. 3A). Knockdown of *Drp1* expression resulted in a reduction of fragmented mitochondria and an increase in the proportion of mitochondria with intermediate and elongated morphologies (Fig. 3B,C). Furthermore, *Drp1* knockdown led to a significant inhibition of the invasive capacity of Huh7-SR and HepG2-SR cells (Fig. 3D). Moreover, the role of *Drp1* in tumor EMT was investigated using the Transwell assay and RT-qPCR analysis. The results indicated that, compared to the control groups, the migrative capacity and the expression levels of *N-cadherin* and *Vimentin* were markedly decreased, whereas the expression of *E-cadherin* and zonula occludens-1 (*ZO-1*) were significantly increased in both Huh7-SR and HepG2-SR cells with *Drp1* knockdown (Fig. 3E,F). To verify the effects of *Drp1 in vivo*, orthotopic xenograft models were established by implanting Huh7-SR and HepG2-SR cells into the livers of mice. Consistent with the RT-qPCR results from the *in vitro* experiments, *Drp1* knockdown impaired EMT progression *in vivo* (Fig. 3G). A similar relationship between *Drp1* and EMT-related proteins was detected in excised liver cancer specimens (Supplementary Fig. S1A–E). Moreover, the total count of lung metastatic lesions in the control group was significantly higher than in the *Drp1* knockdown group (Fig. 3H). The results from these *in vitro* and *in vivo* studies indicated that *Drp1* promoted EMT and metastasis in sorafenib-resistant liver cancer by modulating mitochondrial dynamics.

### 3.4 Mdivi-1 Limits Sorafenib-Resistant Liver Cancer Metastasis by Inhibiting Drp1 Activity

**Figure 4:**
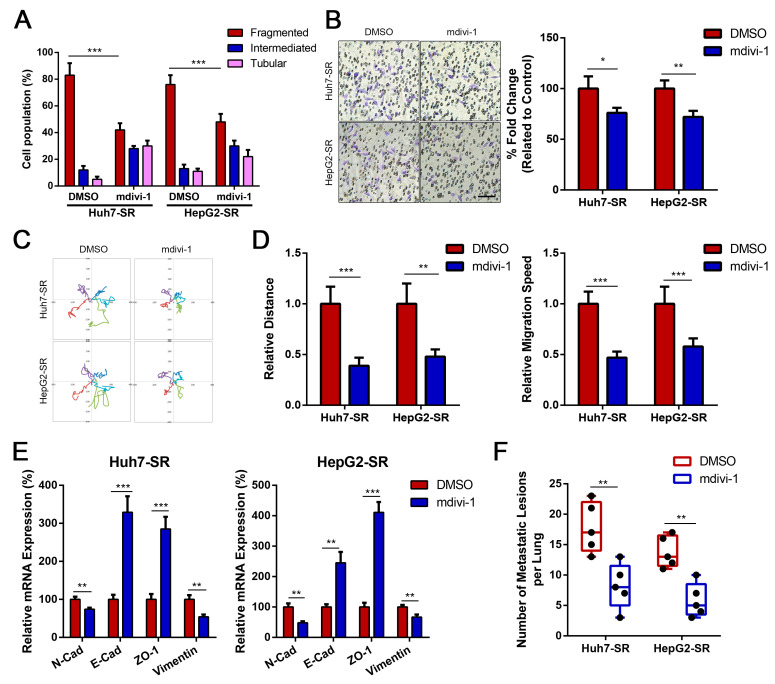
Mdivi-1 limits sorafenib-resistant liver cancer metastasis via inhibiting Drp1 activity. (**A**) Huh7-SR and HepG2-SR cells were treated with 50 μM mdivi-1 or DMSO for 12 h as indicated. The ratio analysis of the morphological changes of mitochondria of 100 cells per group was calculated based on mitochondrial length. Data are presented as the means ± SEM (n = 3/group). (**B**) Representative images of Huh7-SR and HepG2-SR cells invasion in each group in a Transwell system (Left) (Scale bar: 100 μm). Statistical analyses of the number of invasive cells (Right). Data are presented as the means ± SEM (n = 3/group). (**C**) The wind-rose plots for sorafenib-resistant liver cancer cell lines in each show differences in migration. Semi-automated software was used to track cells over time. (**D**) Migration speed and migration distance were measured. Data are presented as the means ± SEM (n = 3/group). (**E**) The mRNA levels of EMT-related genes (including N-cadherin, E-cadherin, ZO-1 and Vimentin) in two groups were detected by RT-qPCR. Data are presented as the means ± SEM (n = 3/group). (**F**) The number of metastatic lesions per nude mouse was calculated. Data are presented as the means ± SEM (n = 5/group). *p < 0.05, **p < 0.01, ***p < 0.001 (all *p* values were obtained by two-sided Student’s *t*-test).

Based on the aforementioned findings, we hypothesized that the administration of mdivi-1, a *Drp1* inhibitor, may represent a therapeutic approach for inhibiting the metastatic ability of sorafenib-resistant liver cancer cells. We confirmed that treatment with mdivi-1 significantly inhibited the activity of Drp1, as reflected by the change of phosphorylated level at Ser616 site of Drp1 (Supplementary Fig. S2). Mdivi-1 treatment markedly increased the number of intermediate and elongated mitochondria and decreased the number of fragmented mitochondria in Huh7-SR and HepG2-SR cells compared to the DMSO control group (Fig. 4A). Transwell invasion assays were performed to assess the invasive capacity of Huh7-SR and HepG2-SR cells in each group. These results collectively demonstrated that the invasive abilities of both Huh7-SR and HepG2-SR cells were reversed to some extent following treatment with mdivi-1 (Fig. 4B). Wind-rose plots also revealed that mdivi-1 treatment notably reduced the migration speed and distance of sorafenib-resistant liver cancer cells (Fig. 4C,D). Furthermore, we examined the changes in EMT-related gene expression in sorafenib-resistant liver cancer cells in these two groups. RT-qPCR analysis demonstrated that mdivi-1 treatment significantly upregulated *E-cadherin* and *ZO-1* mRNA expression and notably downregulated *N-cadherin* and *Vimentin* mRNA expression in both Huh7-SR and HepG2-SR cells compared to the DMSO control groups (Fig. 4E). Finally, an orthotopic xenograft model was established to assess the metastatic ability of sorafenib-resistant liver cancer cells. Fewer lung metastatic lesions were observed in nude mice treated with mdivi-1 compared to the DMSO control group (Fig. 4F). These *in vitro* and *in vivo* studies indicated that mdivi-1 inhibited sorafenib-resistant liver cancer invasion and metastasis by modulating mitochondrial morphology.

### 3.5 Mdivi-1 Promotes Sorafenib-Resistant Liver Cancer Growth in Vitro and in Vivo

**Figure 5:**
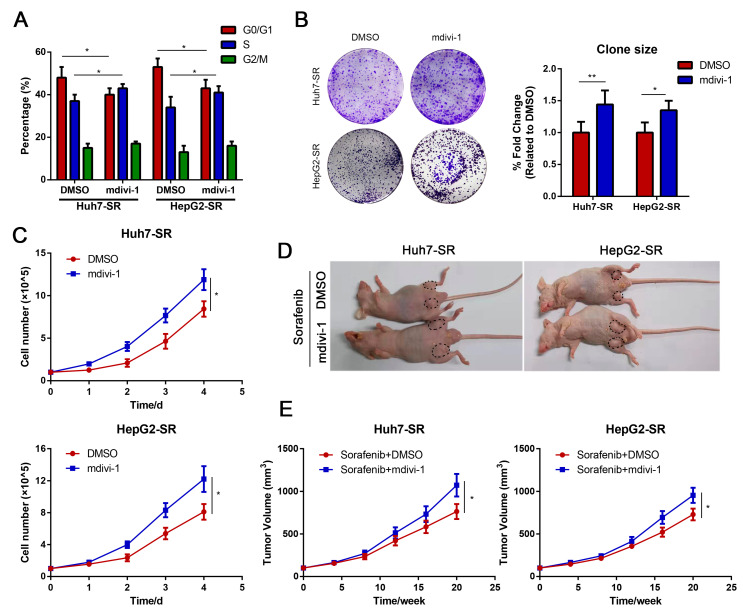
Mdivi-1 promotes liver cancer progression *in vitro* and *in vivo*. (**A**) Huh7-SR and HepG2-SR cells were treated with 50 μM mdivi-1 or DMSO as indicated. Cell cycle assays were performed by staining 7-aminoactinomycin D and analyzing by flow cytometry. (**B**) Images of the plate clone formation assay of Huh7-SR and HepG2-SR cells in two groups. Statistical analyses for the clone size of two liver cancer cell lines in two groups. Data are presented as the means ± SEM (n = 3/group). (**C**) Huh7-SR and HepG2-SR cells in two groups were counted after exposure to treatment according to group design for 0, 1, 2, 3 and 4 d, respectively. Data are presented as the means ± SEM (n = 3/group). (**D**,**E**) A subcutaneous xenograft model was established by implanting Huh7-SR and HepG2-SR cells, respectively. Tumor volume was detected to monitor the tumor group. Data are presented as the means ± SEM (n = 5/group). *p < 0.05, **p < 0.01 (all *p* values were obtained by two-sided Student’s *t*-test).

In further investigations into the role of mdivi-1 in the progression of sorafenib-resistant liver cancer, we interestingly discovered that mdivi-1 stimulated the proliferation of these cancer cells. As shown in Supplementary Fig. S3A,B, flow cytometry analysis revealed that treatment with mdivi-1 did not significantly accelerate the rate of apoptosis in the two sorafenib-resistant liver cancer cell lines. Cell cycle analysis was performed to determine the effect of mdivi-1 on cell cycle regulation, and it was found that mdivi-1 treatment promoted the transition from the G0/G1 phase to the S phase (Fig. 5A). Concurrently, the results of colony formation assays indicated that treatment with mdivi-1 notably increased the colony size of liver cancer cells compared to the control group (Fig. 5B). Furthermore, cell counting assays demonstrated that mdivi-1 significantly promoted the proliferation of liver cancer cells (Fig. 5C). Finally, subcutaneous xenograft models were established by injecting mice with either Huh7-SR or HepG2-SR cells. The average tumor volume in the mdivi-1 treatment group was larger than that in the DMSO control group (Fig. 5D,E). Taken together, these results demonstrated that although mdivi-1 limited the metastatic ability of sorafenib-resistant liver cancer, it promoted the proliferation of these cells.

### 3.6 Mdivi-1 Regulates Glucose Metabolic Reprogramming in Sorafenib-Resistant Liver Cancer Cells

**Figure 6:**
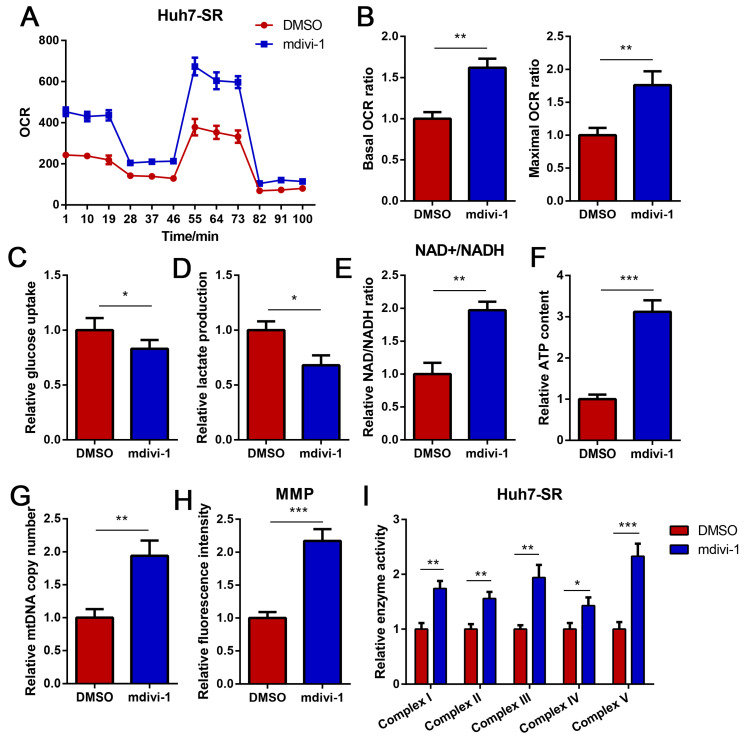
Mdivi-1 regulates glucose metabolic reprogramming in Huh7-SR cells. (**A**) Huh7-SR cells were treated with 50 μM mdivi-1 or DMSO as indicated. The oxygen consumption rate in Huh7-SR cells was measured after treatment according to the group design. (**B**) Statistical analyses of basal OCR ratio and maximal OCR ratio. Data are presented as the means ± SEM (n = 3/group). (**C–F**) The levels of glucose uptake, lactate production, NAD^+^/NADH ratio and ATP content in Huh7-SR cells in each group were detected. Data are presented as the means ± SEM (n = 3/group). (**G**,**H**) The level of mtDNA copy number and mitochondrial membrane potential (MMP) in liver cancer cell line in each group was detected. Data are presented as the means ± SEM (n = 3/group). (**I**) Enzyme activity of respiratory complexes I, II, III, IV and V was detected in liver cancer cell line in each group. Data are presented as the means ± SEM (n = 3/group). *p < 0.05, **p < 0.01, ***p < 0.001 (all *p* values were obtained by two-sided Student’s *t*-test).

Next, we explored whether mdivi-1 could regulate glucose metabolic reprogramming in sorafenib-resistant liver cancer cells, as previous studies indicated that mitochondrial elongation was involved in mediating glucose metabolism reprogramming to maintain tumor cell survival. First, the Seahorse platform was utilized to monitor changes in the metabolic state of Huh7-SR cells in each group. As expected, treatment with mdivi-1 resulted in an increase in both basal and maximal OCR compared to the DMSO control group, suggesting enhanced mitochondrial respiration (Fig. 6A,B). In addition, we found that glucose uptake and lactate production in Huh7-SR cells were significantly decreased after treatment with mdivi-1 (Fig. 6C,D). Given that mitochondria generate ATP through the OXPHOS pathway by utilizing oxygen, we measured the NAD^+^/NADH ratio and ATP production in Huh7-SR cells with or without mdivi-1 treatment. The results showed that mdivi-1 treatment increased the NAD^+^/NADH ratio and cellular ATP production (Fig. 6E,F). This indicated that mdivi-1 could induce a glucose metabolic shift from glycolysis to OXPHOS in sorafenib-resistant liver cancer cells. More importantly, mdivi-1 treatment markedly enhanced the mtDNA copy number and the activity of all five respiratory chain complexes (complexes I-V), and upregulated the mitochondrial membrane potential in sorafenib-resistant liver cancer cells, suggesting that mitochondrial functions in tumor cells were increased by mdivi-1 (Fig. 6G–I). Collectively, these findings indicated that mdivi-1 promoted glucose metabolic reprogramming in sorafenib-resistant liver cancer cells, shifting their metabolism from glycolysis to OXPHOS.

### 3.7 Combination of Mdivi-1 and Iacs-010759 Limits Both Metastatic Ability and Proliferation of Sorafenib-Resistant Liver Cancer Cells in Vitro

**Figure 7:**
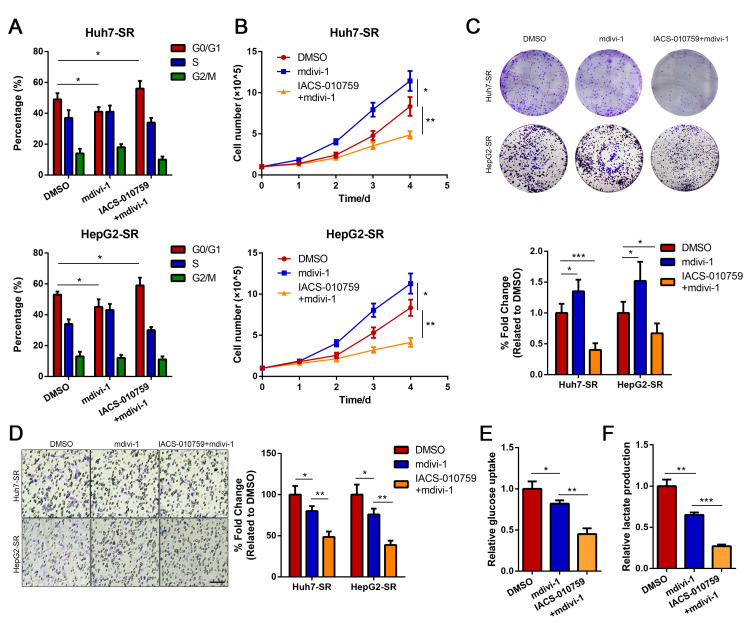
Combination of mdivi-1 and IACS-010759 limits both metastatic and proliferation abilities of liver cancer cells *in vitro*. (**A**) Huh7-SR and HepG2-SR cells were treated with DMSO, 50 μM mdivi-1 and 50 μM mdivi-1 + 100 nM IACS-010759, respectively. Cell cycle assays were performed by staining 7-aminoactinomycin D and analyzing by flow cytometry. (**B**) Huh7-SR and HepG2-SR cells in three groups were counted after exposure to treatment according to group design for 0, 1, 2, 3 and 4 d, respectively. Data are presented as the means ± SEM (n = 3/group). (**C**) Statistical analyses for the clone size of Huh7-SR and HepG2-SR cells in three groups. Data are presented as the means ± SEM (n = 3/group). (**D**) Representative images and statistical analyses of two sorafenib-resistant liver cancer cell lines in each group in a Transwell system (Scale bar: 100 μm). Data are presented as the means ± SEM (n = 3/group). (**E**) The level of glucose uptake was detected. Data are presented as the means ± SEM (n = 3/group). (**F**) The level of lactate production was detected. Data are presented as the means ± SEM (n = 3/group). *p < 0.05, **p < 0.01, ***p < 0.001 (all *p* values were obtained by two-sided Student’s *t*-test).

To address the aforementioned issue of mdivi-1 promoting cell proliferation, we combined IACS-010759, an inhibitor that suppresses OXPHOS energy production, with mdivi-1 to treat sorafenib-resistant liver cancer cells. First, we explored the effect of IACS-010759 on the cell cycle following mdivi-1 treatment. The results demonstrated that cells treated with IACS-010759 exhibited cell cycle arrest at the G1/S phase (Fig. 7A). In addition, we found that the increased proliferation of sorafenib-resistant liver cancer cells following mdivi-1 treatment was significantly reversed after administration of IACS-010759 (Fig. 7B,C). Moreover, the inhibitory effect on the invasive and EMT abilities of sorafenib-resistant liver cancer cells in the mdivi-1 + IACS-010759 group was more pronounced than that observed in the mdivi-1 group alone (Fig. 7D and Supplementary Fig. S4A). Concurrently, we examined the alterations in glucose metabolism in each group. Compared to the control group, treatment with mdivi-1 significantly reduced glucose uptake and lactate accumulation in sorafenib-resistant liver cancer cells, and the addition of IACS-010759 further enhanced these effects (Fig. 7E,F). Furthermore, we demonstrated that IACS-010759 reversed the increase in ATP production and mitochondrial function (as indicated by MMP and mtDNA copy number) observed in sorafenib-resistant liver cancer cells treated with mdivi-1 (Supplementary Fig. S4B–D).

### 3.8 Combination of Mdivi-1 and Iacs-010759 Inhibits Both Proliferation and the Metastatic Ability of Sorafenib-Resistant Liver Cancer Cells in Vivo

To verify the *in vivo* effect of the combined administration of mdivi-1 and IACS-010759, both subcutaneous and orthotopic xenograft models were established. Prior to evaluating the therapeutic efficacy of the combination treatment with mdivi-1 and IACS-010759, we first assessed its biosafety through histomorphological examination and functional evaluation of major organs in healthy mice following administration of mdivi-1, IACS-010759, or their combination. No significant histopathological changes were observed in the heart, lungs, liver, or kidneys across treatment groups (Supplementary Fig. S5A). In addition, comparative analysis of serum biochemical markers—including alanine aminotransferase (ALT), aspartate aminotransferase (AST), creatinine, and creatine kinase—revealed no significant differences among the three groups (Supplementary Fig. S5B).

**Figure 8:**
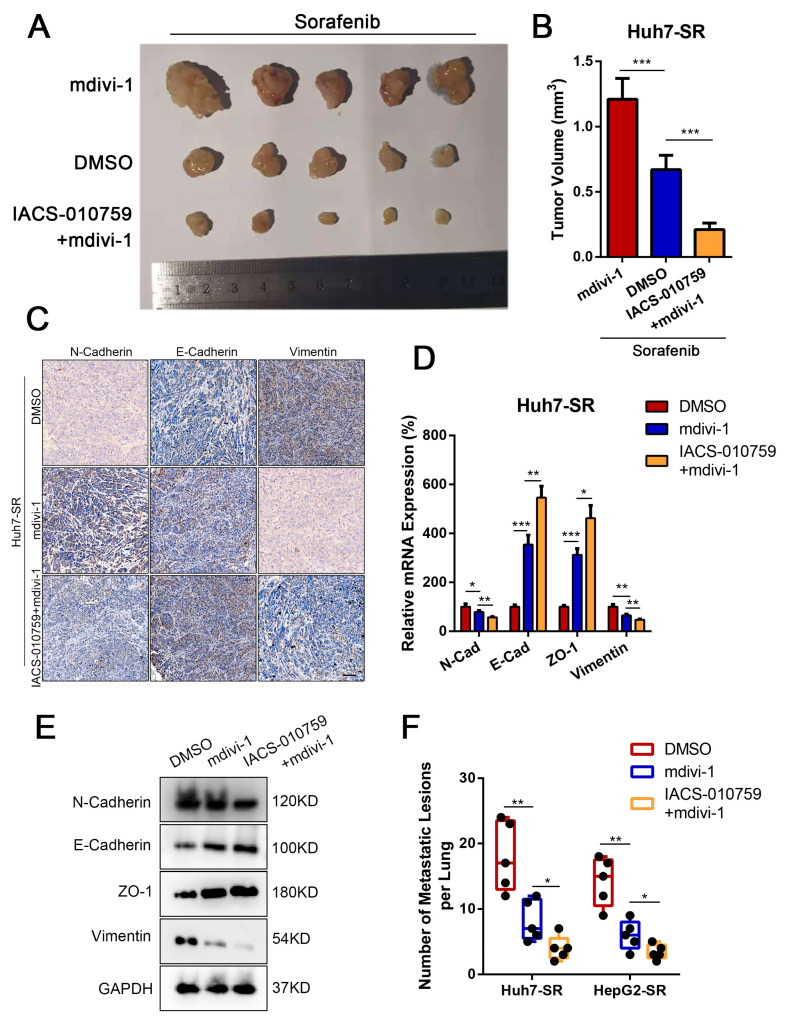
Combination of mdivi-1 and IACS-010759 inhibits both proliferation and metastatic ability of liver cancer cells *in vivo*. (**A**,**B**) A subcutaneous xenograft model was established by implanting Huh7-SR cells and tumor volumes were detected. Data are presented as the means ± SEM (n = 5/group). (**C**–**E**) The mRNA levels of EMT-related genes in Huh7-SR tumor in three groups were detected by immunohistochemical staining (**C**; Scale bar: 100 μm), RT-qPCR (**D**) and western blot assays (**E**). Data are presented as the means ± SEM (n = 5/group). (**F**) The number of metastatic lesions per nude mouse was calculated. Data are presented as the means ± SEM (n = 5/group). *p < 0.05, **p < 0.01, ***p < 0.001 (all *p* values were obtained by two-sided Student’s *t*-test).

The results from the subcutaneous xenograft models demonstrated that the average liver tumor volume in the mdivi-1 + IACS-010759 group was markedly smaller than that in the mdivi-1 group (the largest tumor volume in this study was 1315 mm^3^) and the DMSO control group (Fig. 8A,B), which was consistent with Ki67 staining (Supplementary Fig. S6A,B). Subsequently, tumor tissues from each group were collected, and IHC staining, RT-qPCR, and western blot assays were performed to determine the expression of EMT-related genes. These analyses revealed that, compared to mdivi-1 treatment alone, the combined administration of mdivi-1 and IACS-010759 further upregulated E-cadherin and ZO-1 expression and downregulated N-cadherin and Vimentin expression in liver tumors (Fig. 8C–E). In the orthotopic xenograft models, the number of lung metastatic lesions in the mdivi-1 + IACS-010759 group was lower than that in both the DMSO control and mdivi-1 groups (Fig. 8F). Taken together, these results suggested that the combination of mdivi-1 and IACS-010759 for treating sorafenib-resistant liver cancer cells not only had a comparable effect on inhibiting tumor invasion but also prevented the increased proliferative effect induced by mdivi-1 treatment alone in these cells.

## 4 Discussion

**Figure 9:**
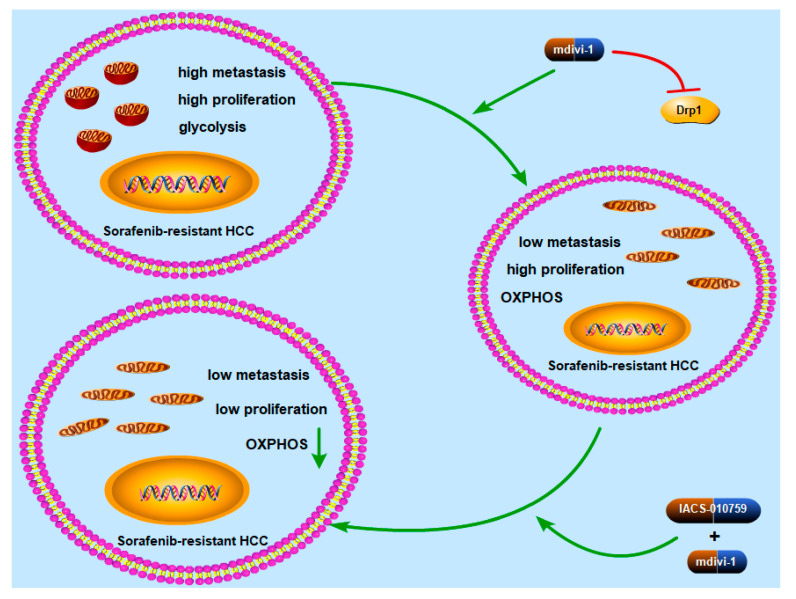
Schematic diagram of a combinative targeting strategy for sorafenib-resistant liver cancer.

Mitochondria are morphologically heterogeneous organelles that frequently undergo changes in shape and subcellular distribution, processes tightly regulated by fusion and fission. Alterations in mitochondrial dynamics directly impact cellular metabolism and have been implicated in cancer initiation, progression, and prognosis [9,20,21]. In this study, we identified Drp1-mediated mitochondrial fission as the predominant mitochondrial phenotype in sorafenib-resistant liver cancer cells, revealing a context-dependent alteration in mitochondrial dynamics associated with drug resistance. Intriguingly, pharmacological inhibition of mitochondrial fission produced a dual and paradoxical effect: while it effectively suppressed invasion and metastasis, it unexpectedly promoted cellular proliferation. This finding highlights a potential therapeutic dilemma and suggests that targeting mitochondrial dynamics alone may be detrimental in this setting. Notably, combined treatment with inhibitors targeting both mitochondrial fission and OXPHOS significantly attenuated both proliferation and metastasis of sorafenib-resistant liver cancer, suggesting that this dual-targeting strategy may represent an effective therapeutic approach for advanced liver cancer (Fig. 9).

Over the years, numerous studies have demonstrated a more fragmented mitochondrial phenotype in various malignant cancer types than in adjacent tissues [22,23]. The mitochondrial network is generally regulated by mitochondrial dynamic proteins, including *Drp1* for fission and *MFN1*, *MFN2*, and *OPA1* for fusion [24]. However, our findings regarding the common mitochondrial morphology and the key proteins involved in liver cancer differed from previous studies, highlighting the complexity of this area. Huang et al. and Zhang et al. reported excessive mitochondrial fission in liver cancer tissues, primarily regulated by an elevated *Drp1* to *MFN1* expression ratio or by MFN1 downregulation, respectively [25]. Conversely, another study found that *MFN1* and *OPA1*-mediated mitochondrial fusion supported liver cancer growth [26]. In addition, it has been demonstrated that mitochondrial elongation resulting from *Drp1* inactivation under energy stress was essential for liver cancer cell survival [27]. In the present study, we observed that sorafenib-resistant liver cancer cell lines exhibited fragmented mitochondria due to *Drp1* upregulation. Genetic modification of *Drp1* expression or pharmacological inhibition of *Drp1* activation markedly impaired the invasive and metastatic capacity of sorafenib-resistant liver cancer *in vitro* and *in vivo*. Previous reports have indicated that persistent mitochondrial fission influences subcellular mitochondrial trafficking and redistribution [28]. Energetically active mitochondria localized to the cortical cytoskeleton near membrane protrusions support membrane dynamics and turnover of focal adhesion complexes, consequently promoting random cell motility [29]. Thus, Drp1-mediated mitochondrial fission and trafficking may play a crucial role in the invasion and metastasis of sorafenib-resistant liver cancer cells, potentially contributing to liver cancer recurrence.

Mdivi-1, a classical *Drp1* inhibitor, has been extensively used in tumor therapy and has been shown to inhibit cellular proliferation and invasion. Meanwhile, administration of mdivi-1 has been reported to reverse cellular apoptosis and promote survival under lethally ischemic or toxic conditions. Therefore, the overall function of mdivi-1 treatment appears to be context-dependent. Our present study revealed that mdivi-1 exhibited two contrasting effects on sorafenib-resistant liver cancer progression: it impaired invasive capacity and triggered cellular proliferation. Following mdivi-1 treatment, mitochondria also became elongated, accompanied by metabolic alterations that supported the growth of sorafenib-resistant liver cancer. In addition, Mitra et al. explored the relationship between mitochondrial dynamics and the cell cycle, finding that mitochondria form a hyperfused and giant network at the G1-S phase transition. Inducing mitochondrial hyperfusion by inhibiting *Drp1* prompted quiescent cells to initiate DNA replication and led to the accumulation of cyclin E, a key regulator of G1-to-S phase progression [30]. Notably, Marsboom et al. [31] suggested that mdivi-1 reduces the proliferation rates of pulmonary artery smooth muscle cells in pulmonary arterial hypertension, likely due to cell cycle arrest in the G2/M phase. However, in our study, mdivi-1 administration to sorafenib-resistant liver cancer cells promoted the transition from the G0/G1 phase to the S phase. These contrasting observations may stem from contrasting effects of mdivi-1 on the cell cycle in malignant tumor cells versus normal smooth muscle cells. Moreover, mitochondrial elongation-mediated glucose metabolism reprogramming is essential for tumor cell survival during energy stress, which aligns with our findings following mdivi-1 treatment. Collectively, these results suggest that targeting mitochondrial dynamics in clinical settings in the future may need to be carefully considered based on the specific cellular context.

DMSO is a common polar aprotic solvent and cryoprotectant composed of a sulfinyl group and two polar methyl groups [32]. These characteristics enable it to dissolve in both aqueous and organic media, allowing it to solubilize a broad range of small molecules, including polar and nonpolar substances. These properties are uniquely crucial for their use as a vehicle for pharmacological compounds in intracellular applications. Therefore, DMSO frequently serves as a control group for testing natural products and related clinical drugs in *in vivo* and *in vitro* experimental studies. DMSO is generally considered a low-toxicity solvent. However, depending on the concentration and cell type, DMSO can affect cell culture. For instance, DMSO at 25% v/v can increase the activity of lysosomal enzymes in rat liver cells [33]. Human peripheral blood lymphocytes treated with 40% v/v DMSO showed increased selectin expression and intracellular activation pathway-related protein phosphorylation [34]. In this study, 1% DMSO was used as the negative control in both *in vitro* and *in vivo* experiments.

The impact of mitochondrial morphology on metabolism has been extensively explored in previous studies [35]. Cancer and stem cells appear to share a common mitochondrial morphology and metabolic profile, characterized by fragmented mitochondria and aerobic glycolysis. This metabolic profile is dependent on genes that regulate mitochondrial fission and fusion [36]. It is widely recognized that persistent mitochondrial fission is indicative of functional immaturity and abnormality. Furthermore, elongated mitochondria facilitate the metabolic transition from glycolysis to OXPHOS, a phenomenon observed in both tumor and non-transformed cells. The underlying mechanisms may involve the facilitation of cristae formation and the assembly of respiratory complexes through mitochondrial elongation, which enhances OXPHOS. This process, in turn, establishes a feedback inhibitory loop on glycolysis via NAD^+^-dependent SIRT1 activation [37]. Furthermore, PGC-1α can be deacetylated by SIRT1, promoting mitochondrial biogenesis and OXPHOS [38]. In addition, mitochondrial fusion enhances the crosstalk between individual mitochondria, leading to decreased mitophagy flux and attenuated damage to mitochondrial DNA, which promotes mitochondrial maturation [39]. Thus, the regulatory relationship between mitochondrial morphology and metabolism warrants further clarification in future studies.

Recently, mitochondrial metabolism has emerged as a therapeutic target for tumor treatment [35]. Although it is widely reported that most tumor cells exhibit a glycolytic phenotype, commonly referred to as the Warburg effect, rather than primarily utilizing the OXPHOS pathway, tumor cells may acquire a stable hybrid metabolic state. In this state, both glycolysis and OXPHOS could be employed concurrently. Targeting both glycolysis and OXPHOS is necessary to overcome metabolic plasticity, representing a novel approach to block the supply of essential substances and energy for tumor growth and invasion [40,41]. In the present study, we found that mdivi-1 administration could inhibit mitochondrial fission and impair the glycolytic phenotype. Combined administration of mdivi-1 with IACS-010759, a clinical-grade small-molecule inhibitor of complex I of the mitochondrial electron transport chain, could synergistically hamper OXPHOS, thereby effectively alleviating the progression of sorafenib-resistant liver cancer. Given that IACS-010759 is currently under evaluation in phase 1 clinical trials for relapsed/refractory acute myeloid leukemia (AML) and solid tumors [42], this combination therapy may hold promising clinical potential.

This study has several limitations. First, the promising preclinical combination of mdivi-1 and IACS-010759 lacks clinical validation. Although IACS-010759 is in Phase 1 trials, the specific combination has not been tested in humans, leaving its efficacy, safety, and off-target effects in sorafenib-resistant liver cancer patients unknown. Second, the use of 1% DMSO as a vehicle control presents a potential confounding variable. Despite its status as a standard low-toxicity control, DMSO can exert subtle, context-dependent biological effects that may influence cellular responses and confound data interpretation.

## 5 Conclusion

In conclusion, our findings indicate that Drp1-mediated mitochondrial fragmentation and a glycolytic phenotype are dominant characteristics of sorafenib-resistant liver cancer cells. This underscores that targeting mitochondrial morphology alone is insufficient for effective tumor suppression and that concurrent metabolic intervention is required. Therefore, a dual-targeting strategy against both mitochondrial dynamics and metabolism may represent a novel and promising therapeutic approach for liver cancer.

## Data Availability

The datasets used and analyzed during the current study are available from the corresponding author on reasonable request.

## Supplementary Materials

The supplementary material is available online at https://www.techscience.com/doi/10.32604/or.2026.067443/s1.

## Abbreviations

*Drp1*	dynamin-related protein 1
*MFN*	mitofusin
*OPA1*	optic atrophy 1
OCR	oxygen consumption rate
OXPHOS	oxidative phosphorylation
DMEM	Dulbecco’s Modified Eagle Medium
FBS	fetal bovine serum
RT-qPCR	reverse transcription-quantitative PCR
IHC	immunohistochemical
SR	sorafenib-resistant
EMT	epithelial-mesenchymal transition

## References

[1] Siegel RL , Miller KD , Fuchs HE , Jemal A . Cancer statistics, 2022. CA Cancer J Clin. 2022; 72( 1): 7– 33. doi:10.3322/caac.21708. 35020204

[2] Kulik L , El-Serag HB . Epidemiology and management of hepatocellular carcinoma. Gastroenterology. 2019; 156( 2): 477– 91. doi:10.1053/j.gastro.2018.08.065. 30367835 PMC6340716

[3] Toh MR , Wong EY , Wong SH , Ng AW , Loo LH , Chow PK , et al. Global epidemiology and genetics of hepatocellular carcinoma. Gastroenterology. 2023; 164( 5): 766– 82. doi:10.1053/j.gastro.2023.01.033. 36738977

[4] Li CX , Wang JS , Wang WN , Xu DK , Zhou YT , Sun FZ , et al. Expression dynamics of periodic transcripts during cancer cell cycle progression and their correlation with anticancer drug sensitivity. Mil Med Res. 2022; 9( 1): 71. doi:10.1186/s40779-022-00432-w. 36529792 PMC9762028

[5] Lin Y , Zhang F , Guo W , Guo J , Qiu X , Sun Y , et al. Hyperbaric oxygen targets RCN1 to modulate ER-mitochondria crosstalk and ameliorate sorafenib resistance in hepatocellular carcinoma. Drug Resist Updates. 2025; 85: 101342. doi:10.1016/j.drup.2025.101342. 41385809

[6] Wang Q , Zhang P , Li Z , Feng X , Lv C , Zhang H , et al. Evaluation of polymer nanoformulations in hepatoma therapy by established rodent models. Theranostics. 2019; 9( 5): 1426. doi:10.7150/thno.31683. 30867842 PMC6401493

[7] Tang W , Chen Z , Zhang W , Cheng YE , Zhang B , Wu F , et al. The mechanisms of sorafenib resistance in hepatocellular carcinoma: Theoretical basis and therapeutic aspects. Signal Transduct Target Ther. 2020; 5( 1): 87. doi:10.1038/s41392-020-0187-x. 32532960 PMC7292831

[8] Wu FQ , Fang T , Yu LX , Lv GS , Lv HW , Liang D , et al. ADRB2 signaling promotes HCC progression and sorafenib resistance by inhibiting autophagic degradation of HIF1alpha. J Hepatol. 2016; 65( 2): 314– 24. doi:10.1016/j.jhep.2016.04.019. 27154061

[9] Bock FJ , Tait SWG . Mitochondria as multifaceted regulators of cell death. Nat Rev Mol Cell Biol. 2020; 21( 2): 85– 100. doi:10.1038/s41580-019-0173-8. 31636403

[10] Gao S , Hu J . Mitochondrial fusion: The machineries in and out. Trends Cell Biol. 2021; 31( 1): 62– 74. doi:10.1016/j.tcb.2020.09.008. 33092941

[11] Zhang K , Zhang W , Zhang L , Hou X , Tian R , Hu Z , et al. OPA1 mutations in dominant optic atrophy: Domain-specific defects in mitochondrial fusion and apoptotic regulation. J Transl Med. 2025; 23( 1): 471. doi:10.1186/s12967-025-06471-w. 40275276 PMC12020257

[12] Jin P , Jiang J , Zhou L , Huang Z , Nice EC , Huang C , et al. Mitochondrial adaptation in cancer drug resistance: Prevalence, mechanisms, and management. J Hematol Oncol. 2022; 15( 1): 97. doi:10.1186/s13045-022-01313-4. 35851420 PMC9290242

[13] Martínez-Reyes I , Chandel NS . Cancer metabolism: Looking forward. Nat Rev Cancer. 2021; 21( 10): 669– 80. doi:10.1038/s41568-021-00378-6. 34272515

[14] Feng J , Li J , Wu L , Yu Q , Ji J , Wu J , et al. Emerging roles and the regulation of aerobic glycolysis in hepatocellular carcinoma. J Exp Clin Cancer Res. 2020; 39( 1): 126. doi:10.1186/s13046-020-01629-4. 32631382 PMC7336654

[15] Zhang J , Han ZQ , Wang Y , He QY . Alteration of mitochondrial protein succinylation against cellular oxidative stress in cancer. Mil Med Res. 2022; 9( 1): 6. doi:10.1186/s40779-022-00367-2. 35115046 PMC8815146

[16] Delaunay S , Pascual G , Feng B , Klann K , Behm M , Hotz-Wagenblatt A , et al. Mitochondrial RNA modifications shape metabolic plasticity in metastasis. Nature. 2022; 607( 7919): 593– 603. doi:10.1038/s41586-022-04898-5. 35768510 PMC9300468

[17] Zheng J , Li H , He L , Huang Y , Cai J , Chen L , et al. Preconditioning of umbilical cord-derived mesenchymal stem cells by rapamycin increases cell migration and ameliorates liver ischaemia/reperfusion injury in mice via the CXCR4/CXCL12 axis. Cell Prolif. 2019; 52( 2): e12546. doi:10.1111/cpr.12546. 30537044 PMC6496237

[18] Zheng J , Chen L , Lu T , Zhang Y , Sui X , Li Y , et al. MSCs ameliorate hepatocellular apoptosis mediated by PINK1-dependent mitophagy in liver ischemia/reperfusion injury through AMPKα activation. Cell Death Dis. 2020; 11( 4): 256. doi:10.1038/s41419-020-2424-1. 32312955 PMC7171190

[19] Anderson GR , Wardell SE , Cakir M , Yip C , Ahn YR , Ali M , et al. Dysregulation of mitochondrial dynamics proteins are a targetable feature of human tumors. Nat Commun. 2018; 9( 1): 1677. doi:10.1038/s41467-018-04033-x. 29700304 PMC5919970

[20] Kenny TC , Kıvanç B . Mitochondria and cancer. Cold Spring Harb Perspect Med. 2024; 14( 12): a041534. doi:10.1101/cshperspect.a041534. 38692736 PMC11610758

[21] Borcherding N , Brestoff JR . The power and potential of mitochondria transfer. Nature. 2023; 623( 7986): 283– 91. doi:10.1038/s41586-023-06537-z. 37938702 PMC11590279

[22] Kenny TC , Gomez ML , Germain D . Mitohormesis, UPR^mt^, and the complexity of mitochondrial DNA landscapes in cancer. Cancer Res. 2019; 79( 24): 6057– 66. doi:10.1158/0008-5472.CAN-19-1395. 31484668 PMC6983317

[23] Zheng X , Qian Y , Fu B , Jiao D , Jiang Y , Chen P , et al. Mitochondrial fragmentation limits NK cell-based tumor immunosurveillance. Nat Immunol. 2019; 20( 12): 1656– 67. doi:10.1038/s41590-019-0511-1. 31636463

[24] Vodicka P , Vodenkova S , Danesova N , Vodickova L , Zobalova R , Tomasova K , et al. Mitochondrial DNA damage, repair, and replacement in cancer. Trends Cancer. 2025; 11( 1): 62– 73. doi:10.1016/j.trecan.2024.09.010. 39438191

[25] Li S , Han S , Zhang Q , Zhu Y , Zhang H , Wang J , et al. FUNDC2 promotes liver tumorigenesis by inhibiting MFN1-mediated mitochondrial fusion. Nat Commun. 2022; 13( 1): 3486. doi:10.1038/s41467-022-31187-6. 35710796 PMC9203792

[26] Li M , Wang L , Wang Y , Zhang S , Zhou G , Lieshout R , et al. Mitochondrial fusion via OPA1 and MFN1 supports liver tumor cell metabolism and growth. Cells. 2020; 9( 1): 121. doi:10.3390/cells9010121. 31947947 PMC7017104

[27] Li R , Wang Z , Cheng L , Cheng Z , Wu Q , Chen F , et al. Coordination of SLC39A1 and DRP1 facilitates HCC recurrence by impairing mitochondrial quality control. Clin Transl Med. 2025; 15( 5): e70362. doi:10.1002/ctm2.70362. 40462487 PMC12134400

[28] Agarwal E , Altman BJ , Ho Seo J , Bertolini I , Ghosh JC , Kaur A , et al. Myc regulation of a mitochondrial trafficking network mediates tumor cell invasion and metastasis. Mol Cell Biol. 2019; 39( 14): e00109– 19. doi:10.1128/MCB.00109-19. 31061095 PMC6597883

[29] Seo JH , Agarwal E , Bryant KG , Caino MC , Kim ET , Kossenkov AV , et al. Syntaphilin ubiquitination regulates mitochondrial dynamics and tumor cell movements. Cancer Res. 2018; 78( 15): 4215– 28. doi:10.1158/0008-5472.CAN-18-0595. 29898993 PMC6072605

[30] Tábara LC , Segawa M , Prudent J . Molecular mechanisms of mitochondrial dynamics. Nat Rev Mol Cell Biol. 2025; 26( 2): 123– 46. doi:10.1038/s41580-024-00785-1. 39420231

[31] Marsboom G , Toth PT , Ryan JJ , Hong Z , Wu X , Fang YH , et al. Dynamin-related protein 1–mediated mitochondrial mitotic fission permits hyperproliferation of vascular smooth muscle cells and offers a novel therapeutic target in pulmonary hypertension. Circ Res. 2012; 110( 11): 1484– 97. doi:10.1161/CIRCRESAHA.111.263848. 22511751 PMC3539779

[32] Miralles-Robledillo JM , Torregrosa-Crespo J , Martínez-Espinosa RM , Pire C . DMSO reductase family: Phylogenetics and applications of extremophiles. Int J Mol Sci. 2019; 20( 13): 3349. doi:10.3390/ijms20133349. 31288391 PMC6650914

[33] Misch DW , Misch MS . Dimethyl sulfoxide: Activation of lysosomes *in vitro*. Proc Natl Acad Sci U S A. 1967; 58( 6): 2462– 7. doi:10.1073/pnas.58.6.2462. 5242219 PMC223858

[34] Wedner HJ , Bass G . Induction of the tyrosine phosphorylation of a 66 kd soluble protein by DMSO in human peripheral blood T lymphocytes. Biochem Biophys Res Commun. 1986; 140( 2): 743– 9. doi:10.1016/0006-291X(86)90794-1. 3490851

[35] Du H , Xu T , Yu S , Wu S , Zhang J . Mitochondrial metabolism and cancer therapeutic innovation. Signal Transduct Target Ther. 2025; 10( 1): 245. doi:10.1038/s41392-025-02311-x. 40754534 PMC12319113

[36] Raggi C , Taddei ML , Rae C , Braconi C , Marra F . Metabolic reprogramming in cholangiocarcinoma. J Hepatol. 2022; 77( 3): 849– 64. doi:10.1016/j.jhep.2022.04.038. 35594992

[37] Zhu S , Dong Z , Ke X , Hou J , Zhao E , Zhang K , et al. The roles of sirtuins family in cell metabolism during tumor development. Semin Cancer Biol. 2018; 57: 59– 71. doi:10.1016/j.semcancer.2018.11.003. 30453040

[38] Wu YX , Yang XY , Han BS , Hu YY , An T , Lv BH , et al. Naringenin regulates gut microbiota and SIRT1/PGC-1α signaling pathway in rats with letrozole-induced polycystic ovary syndrome. Biomed Pharmacother. 2022; 153: 113286. doi:10.1016/j.biopha.2022.113286. 35724506

[39] Yamada T , Ikeda A , Murata D , Wang H , Zhang C , Khare P , et al. Dual regulation of mitochondrial fusion by Parkin–PINK1 and OMA1. Nature. 2025; 639( 8055): 776– 83. doi:10.1038/s41586-025-08590-2. 39972141 PMC12399782

[40] Jia D , Park JH , Jung KH , Levine H , Kaipparettu BA . Elucidating the metabolic plasticity of cancer: Mitochondrial reprogramming and hybrid metabolic states. Cells. 2018; 7( 3): 21. doi:10.3390/cells7030021. 29534029 PMC5870353

[41] Jia D , Lu M , Jung KH , Park JH , Yu L , Onuchic JN , et al. Elucidating cancer metabolic plasticity by coupling gene regulation with metabolic pathways. Proc Natl Acad Sci U S A. 2019; 116( 9): 3909– 18. doi:10.1073/pnas.1816391116. 30733294 PMC6397570

[42] Molina JR , Sun Y , Protopopova M , Gera S , Bandi M , Bristow C , et al. An inhibitor of oxidative phosphorylation exploits cancer vulnerability. Nat Med. 2018; 24( 7): 1036– 46. doi:10.1038/s41591-018-0052-4. 29892070

