# Supplementary material for: Targeting Dynamin-Related Protein 1 and Glucose Metabolism Reverses Acquired Resistance to Sorafenib in Liver Cancer
Source: Oncol Res. 2026 Jun 16;34(7):21. doi: 10.32604/or.2026.067443 (PMC13292058; doi:10.32604/or.2026.067443)
Supplement: Supplementary file 1 [file OncolRes-34-67443-s001.zip › Supplementary_Figures_S1-S6.docx]

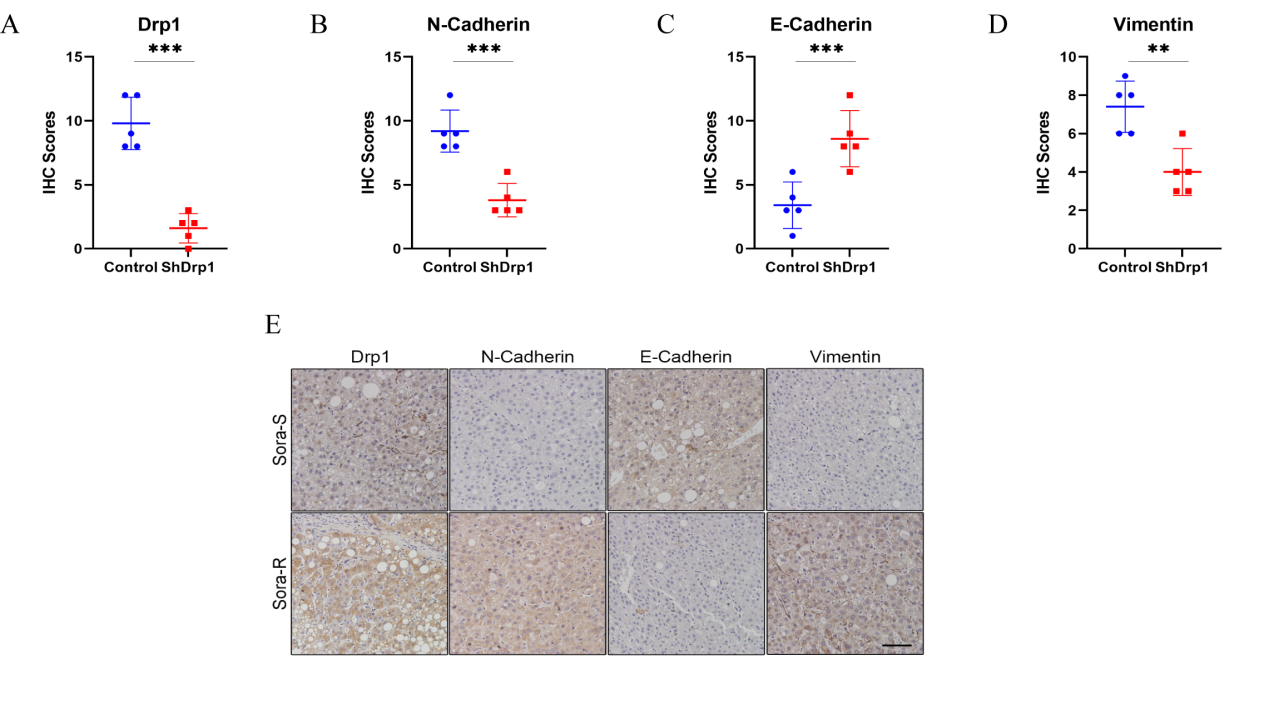


**Figure S1.** The relationship between Drp1 and EMT-related proteins was detected in excised liver cancer specimens. (A-D) IHC analysis of the *Drp1*, N-Cadherin, E-Cadherin, and Vimentin in the control group and the Sh*Drp1* group. ***p*<0.01, ****p*<0.001 (all *p* values were obtained by two-sided Student’s *t* test). (E) Representative images of immunohistochemical staining of Drp1 and EMT-related genes. Scale bar: 100 μm.


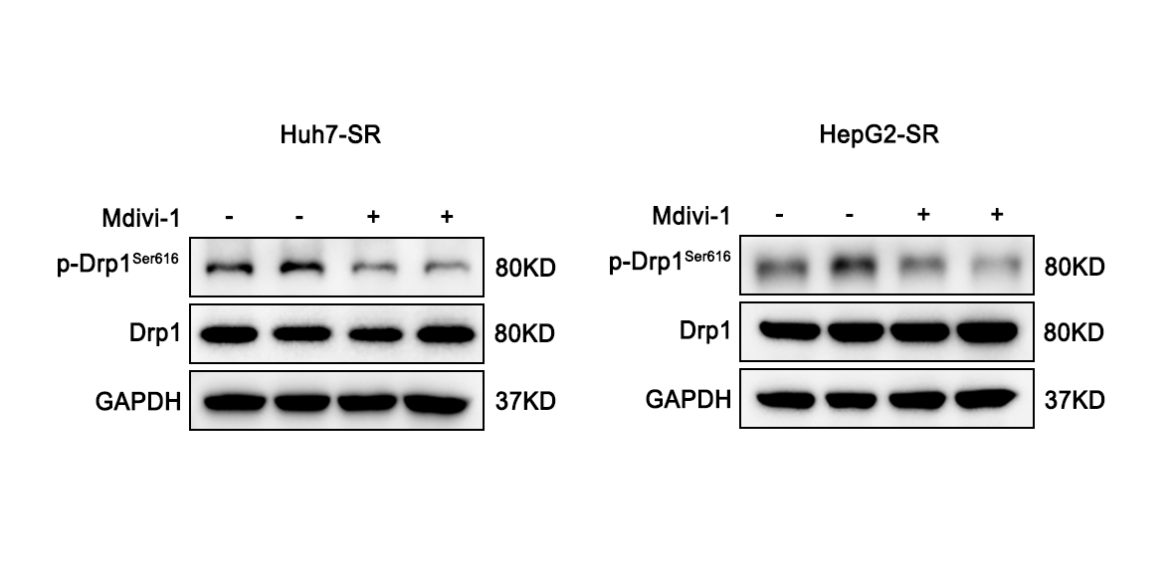


**Figure S2.** Western blotting showed the levels of p-Drp1ser616 and Drp1 in each group.


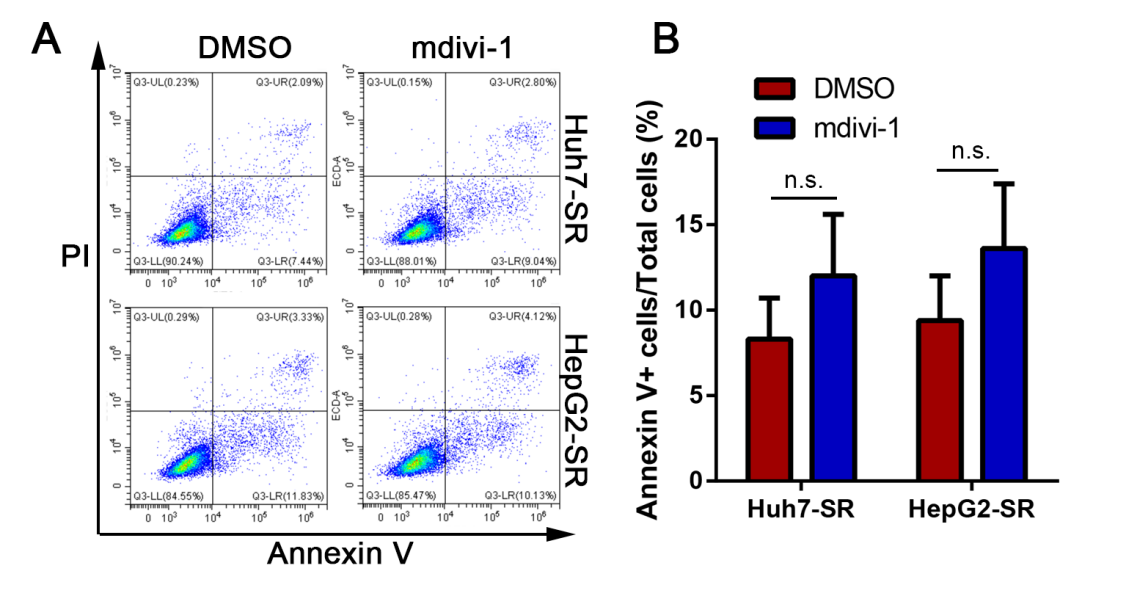


**Figure S3.** Flow cytometry was used to analyze the apoptotic rate of sorafenib-resistant liver cancer cell lines in each group. (A-B) Cell apoptosis was measured by the flow cytometric detection of Annexin V/PI. Data are presented as the means ± SEM (n=3/group). n.s., no significance.


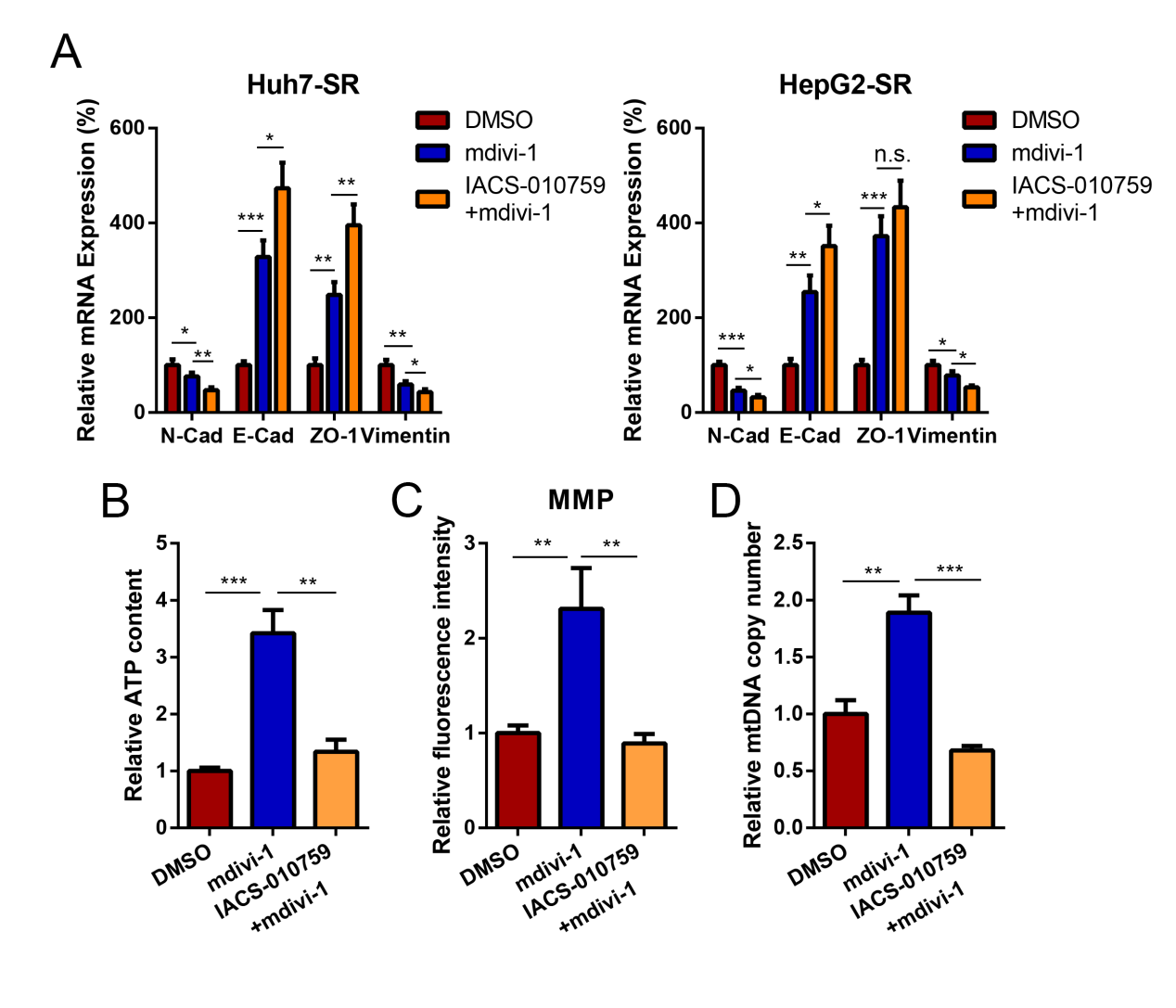


**Figure S4.** Analysis of EMT-related gene expression, ATP levels and mitochondrial function in each group. (A) The mRNA levels of EMT-related genes (including N-cadherin, E-cadherin, ZO-1 and Vimentin) in each group were detected by RT-qPCR. Data are presented as the means ± SEM (n=3/group). (B-D) The level of ATP content (B), mitochondrial membrane potential (C) and mtDNA copy number (D) in Huh7-SR cells in each group was detected. Data are presented as the means ± SEM (n=3/group). n.s., no significance, **p*<0.05, ***p*<0.01, ****p*<0.001 (all *p* values were obtained by two-sided Student’s *t* test).


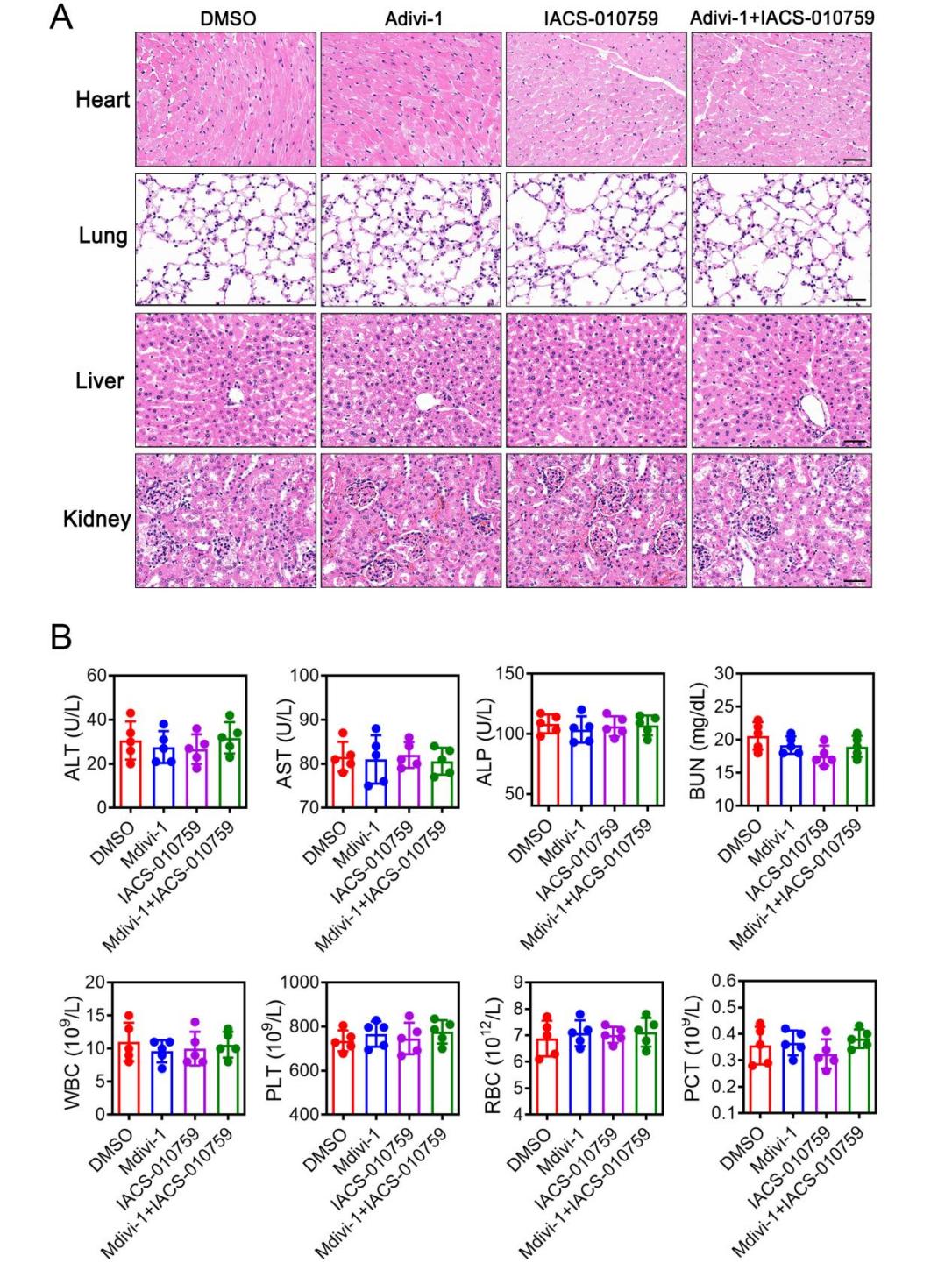


**Figure S5**: Safety evaluation of combination treatment in mice. (A) Representative H&E staining of the liver, kidney, heart, and lung sections from mice treated with pevonedistat or vehicle control. Scale bar = 50 μm. (B) Serum levels of alanine aminotransferase (ALT), aspartate aminotransferase (AST), creatine kinase (CK), and urea in mice treated with pevonedistat compared to vehicle control. Data are presented as mean ± SD (n = 5 per group). Statistical significance was determined by a two-tailed Student’s *t*-test or one-way ANOVA.


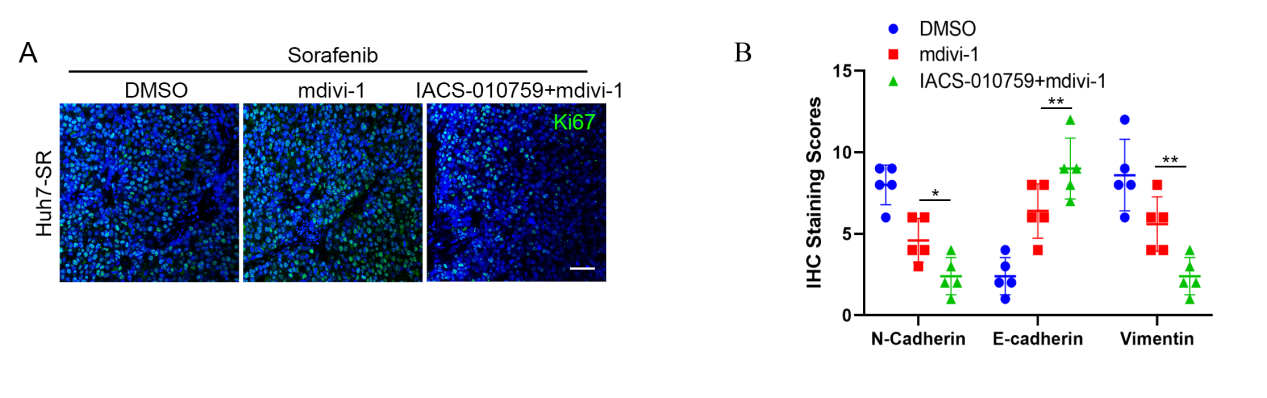


**Figure S6:** Ki67 proliferation and EMT marker expression in each group. (A) Cell proliferation was measured by Ki67 staining (Scale bar: 100 μm). (B) IHC analysis of the N-Cadherin, E-Cadherin, and Vimentin in the DMSO group, mdivi-1 and IACS-010759+mdivi-1 group, **p*<0.05, ***p*<0.01 (all *p* values were obtained by two-sided Student’s *t* test).
