# Supplementary material for: Targeting Dynamin-Related Protein 1 and Glucose Metabolism Reverses Acquired Resistance to Sorafenib in Liver Cancer
Source: Oncol Res. 2026 Jun 16;34(7):21. doi: 10.32604/or.2026.067443 (PMC13292058; doi:10.32604/or.2026.067443)
Supplement: Supplementary file 1 [file OncolRes-34-67443-s001.zip › Supplementary_Table_S1.docx]

**Supplementary Table S1. Primer sequences for quantitative RT-PCR**

| Gene Symbol | Sequence direction | Sequence |
| --- | --- | --- |
| N-Cad | Forward primer | 5’-CCTCCAGAGTTTACTGCCATGAC-3’ |
|  | Reverse primer | 5’-GTAGGATCTCCGCCACTGATTC-3’ |
| E-Cad | Forward primer | 5’-GCCTCCTGAAAAGAGAGTGGAAG-3’ |
|  | Reverse primer | 5’-TGGCAGTGTCTCTCCAAATCCG-3’ |
| ZO-1 | Forward primer | 5’-GTCCAGAATCTCGGAAAAGTGCC-3’ |
|  | Reverse primer | 5’-CTTTCAGCGCACCATACCAACC-3’ |
| Vimentin | Forward primer | 5’-AGGCAAAGCAGGAGTCCACTGA-3’ |
|  | Reverse primer | 5’-ATCTGGCGTTCCAGGGACTCAT-3’ |
| ACTN | Forward primer | 5’-CACCATTGGCAATGAGCGGTTC-3’ |
|  | Reverse primer | 5’-AGGTCTTTGCGGATGTCCACGT-3’ |
| mtDNA (ND-1) | Forward primer | 5’-CCCTAAAACCCGCCACATCT-3’ |
|  | Reverse primer | 5’-GAGCGATGGTGAGAGCTAAGGT-3’ |
