# Supplementary figures and images for: Targeting Dynamin-Related Protein 1 and Glucose Metabolism Reverses Acquired Resistance to Sorafenib in Liver Cancer
Source: Oncol Res. 2026 Jun 16;34(7):21. doi: 10.32604/or.2026.067443 (PMC13292058; doi:10.32604/or.2026.067443)

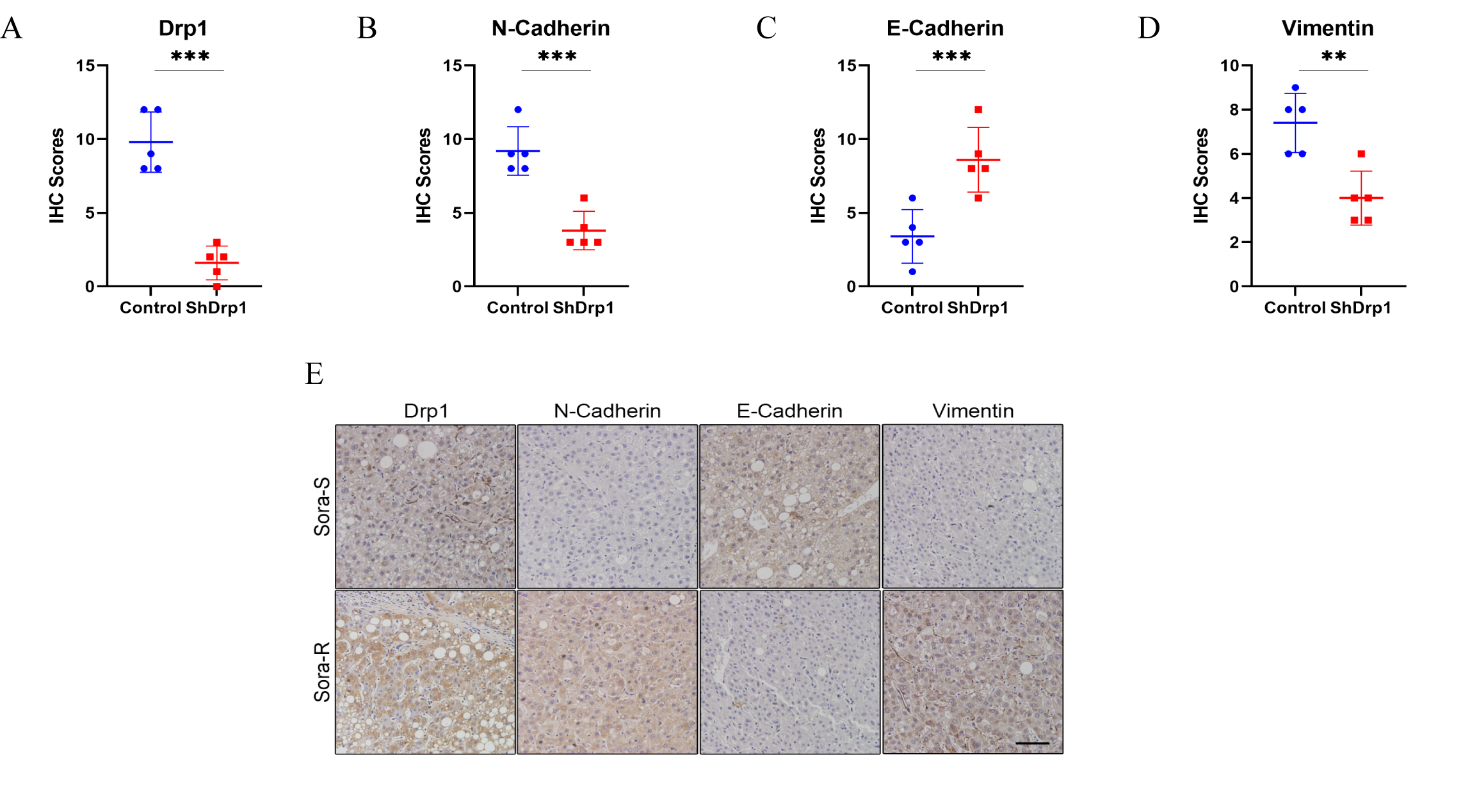

Supplement: Supplementary file 1 [file OncolRes-34-67443-s001.zip › Figure_S1.tif]

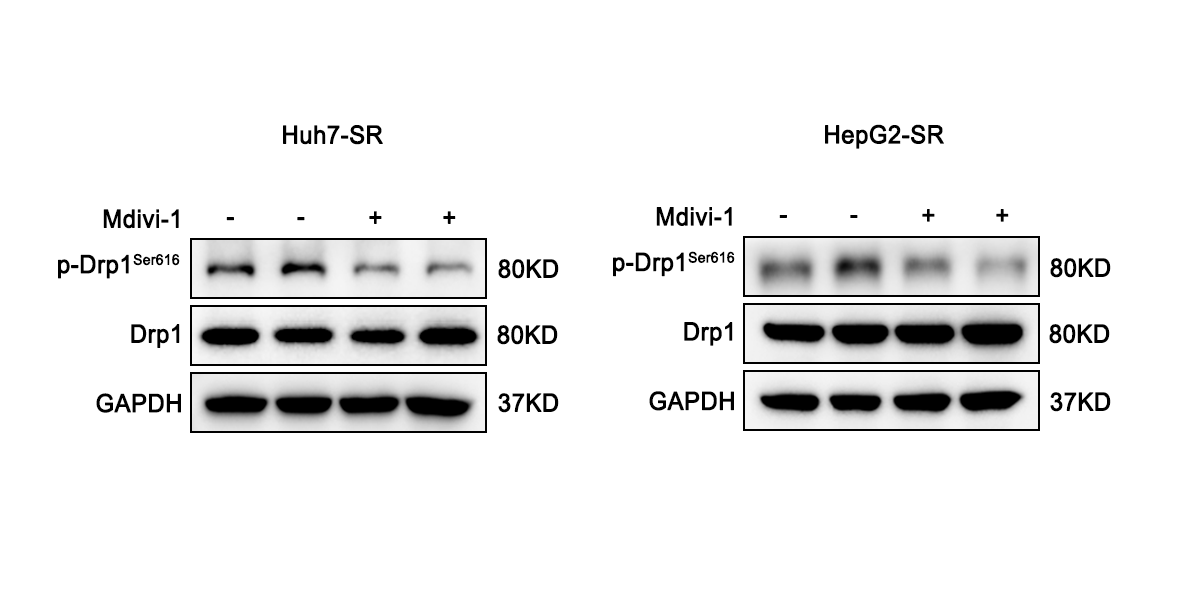

Supplement: Supplementary file 1 [file OncolRes-34-67443-s001.zip › Figure_S2.tif]

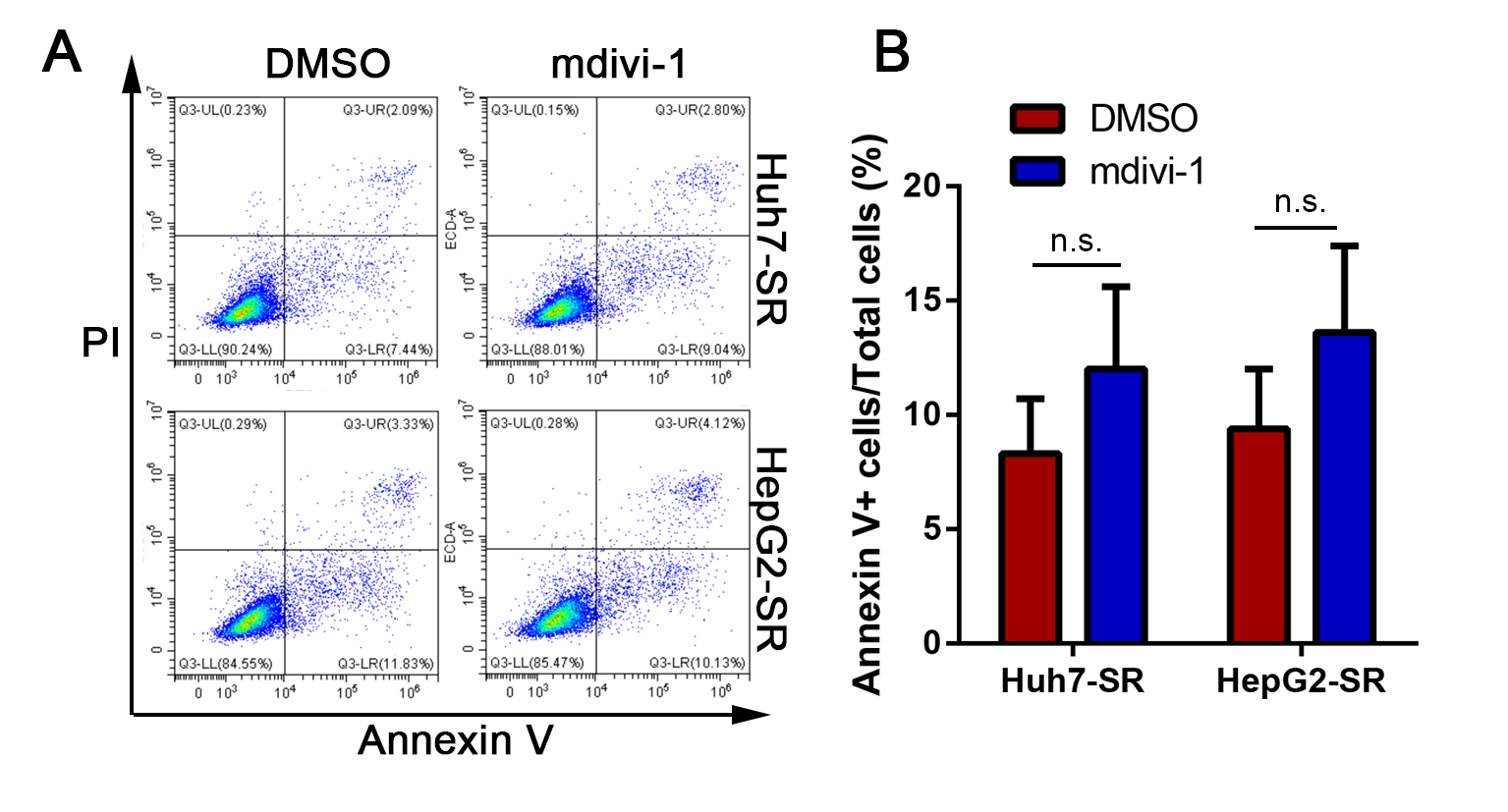

Supplement: Supplementary file 1 [file OncolRes-34-67443-s001.zip › Figure_S3.tif]

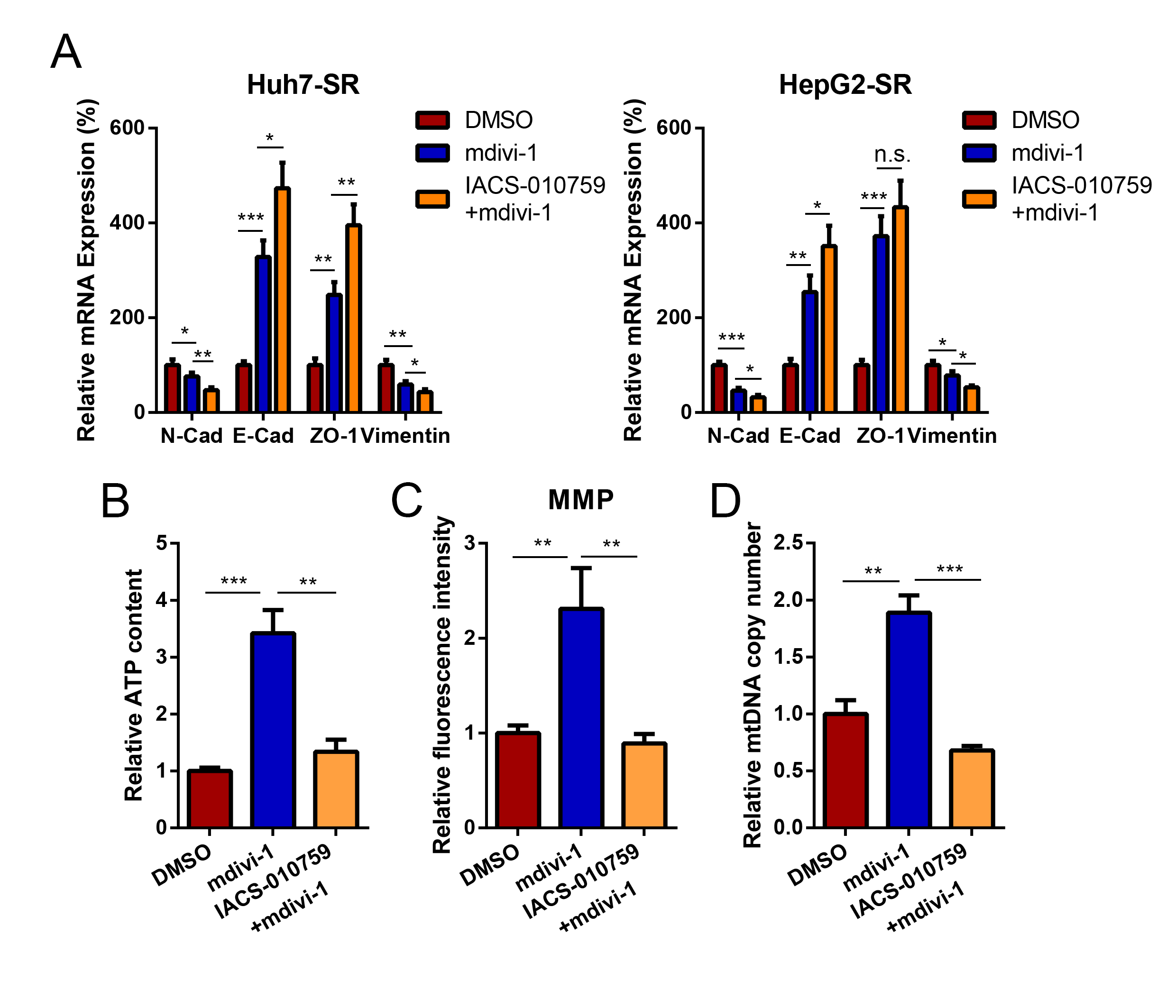

Supplement: Supplementary file 1 [file OncolRes-34-67443-s001.zip › Figure_S4.tif]

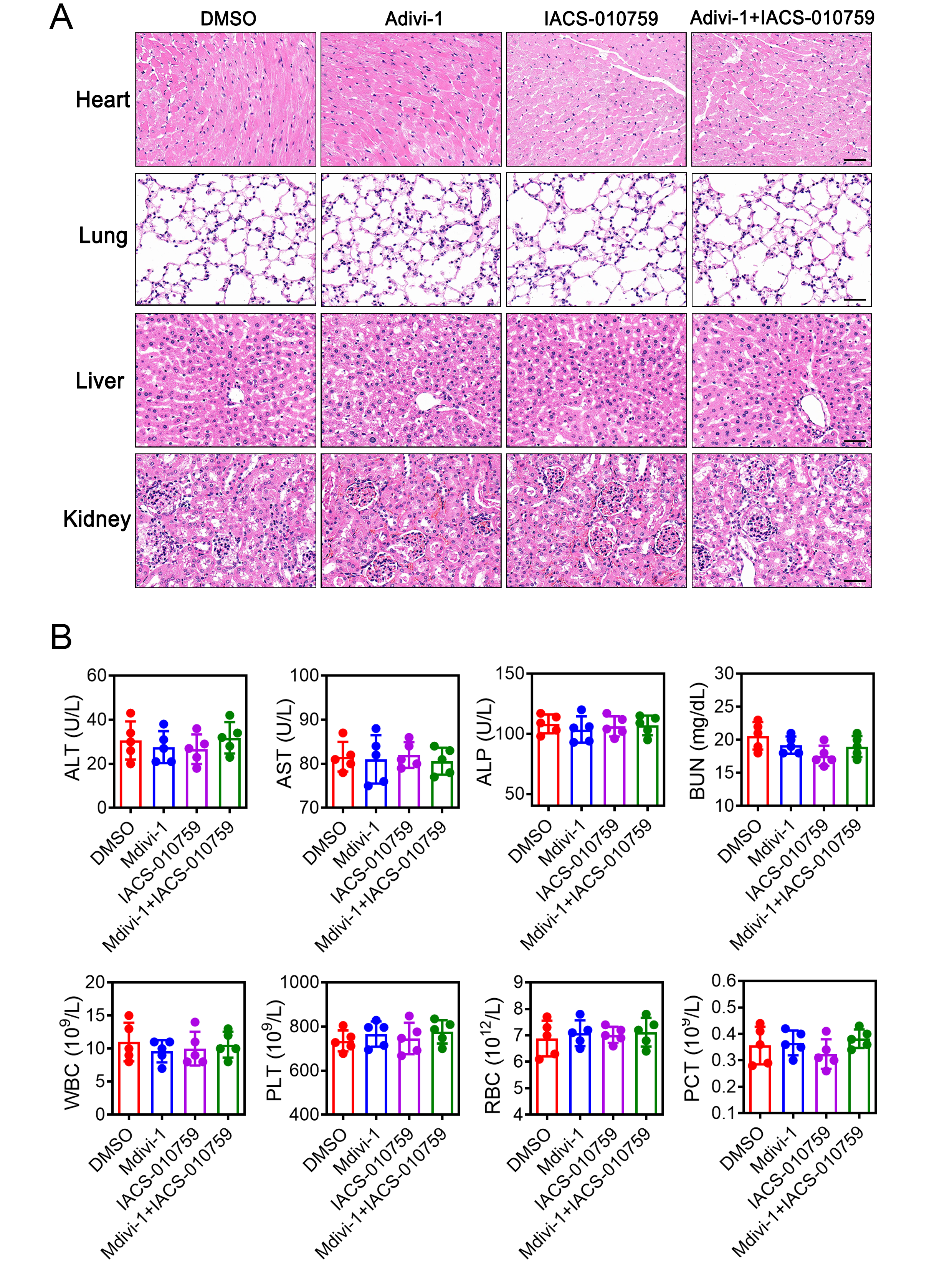

Supplement: Supplementary file 1 [file OncolRes-34-67443-s001.zip › Figure_S5.tif]

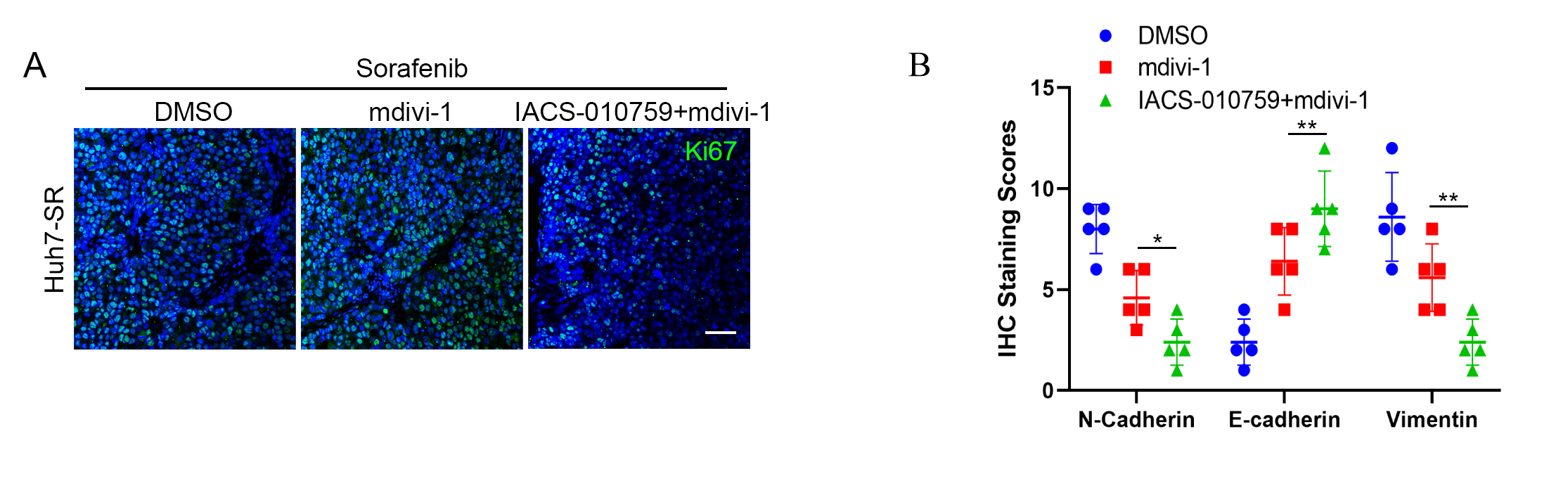

Supplement: Supplementary file 1 [file OncolRes-34-67443-s001.zip › Figure_S6.tif]
